# In Situ Chemical Modulation of Graphitization Degree of Carbon Fibers and Its Potassium Storage Mechanism

**DOI:** 10.1002/advs.202401292

**Published:** 2024-04-01

**Authors:** Shuangsheng Xiong, Qi Wu, Yuan Gao, Zhiping Li, Chen Wang, Shuo Wang, Zheng Li, Li Hou, Faming Gao

**Affiliations:** ^1^ Hebei Key Laboratory of Applied Chemistry State Key Laboratory of Metastable Materials Science and Technology Yanshan University Qinhuangdao 066004 China; ^2^ College of Chemical Engineering and Materials Science Tianjin University of Science and Technology Tianjin 300457 China

**Keywords:** graphite anode, K^+^ storage mechanism, modulation of graphitization degree, potassium ion batteries (PIBs), solid electrolyte interphase (SEI) film

## Abstract

Graphite is considered to be the most auspicious anode candidate for potassium ion batteries. However, the inferior rate performances and cycling stability restrict its practical applications. Few studies have investigated the modulating the graphitization degree of graphitic materials. Herein, a nitrogen‐doped carbon‐coated carbon fiber composite with tunable graphitization (CNF@NC) through etching growth, in‐situ oxidative polymerization, and subsequent carbonization process is reported. The prepared CNF@NC with abundant electrochemical active sites and a rapid K^+^/electron transfer pathway, can effectively shorten the K^+^ transfer distance and promote the rapid insertion/removal of K^+^. Amorphous domains and short‐range curved graphite layers can provide ample mitigation spaces for K^+^ storage, alleviating the volume expansion of the highly graphitized CNF during repeated K^+^ insertion/de‐intercalation. As expected, the CNF@NC‐5 electrode presents a high initial coulombic efficiency (ICE) of 69.3%, an unprecedented reversible volumetric capacity of 510.2 mA h cm^−3^ at 0.1 A g^−1^ after 100 cycles with the mass‐capacity of 294.9 mA h g^−1^. The K^+^ storage mechanism and reaction kinetic analysis are studied by combining in‐situ analysis and first‐principles calculation. It manifests that the K^+^ storage mechanism in CNF@NC‐5 is an adsorption‐insertion‐insertion mechanism (i.e., the “1+2” model). The solid electrolyte interphase (SEI) film forming is also detected.

## Graphical abstract

A series of CNF@NC‐x composites are successfully prepared, confirming the feasibility of in situ chemical modulation of the degree of CNF graphitization. Meanwhile, a “1+2” model K^+^ storage mechanism is proposed and verified by in situ characterizations, and the formation of SEI layer is also observed using in situ Raman spectroscopy.

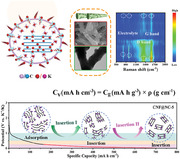

## 1 Introduction

Graphite, as a successful commercial anode material for lithium‐ion batteries (LIBs), is deemed to be the most up‐and‐coming anode candidate for potassium‐ion batteries (PIBs).^[^ ^1^ ^]^ Unlike sodium‐ion batteries (SIBs) forming NaC_64_ compound with an extremely low theoretical capacity (≈35 mA h g^−1^),^[^ ^2^ ^]^ K^+^ can electrochemically insert/extract in the graphite layer to generate K‐graphite intercalation compound (KC_8_), which achieved a considerable theoretical specific capacity (279 mA h g^−1^, only 30% lower than LIBs)^[^ ^3^ ^]^ and slow charge/discharge plateau (<0.2 V).^[^ ^4^ ^]^ Notwithstanding, the conventional graphite anode, seeing its limited interlayer spacing (3.35 Å), long diffusion paths (a few micrometers), and sluggish reaction kinetics, suffers from humongous volume changes (≈61%)^[^ ^3^, ^4^, ^5^ ^]^ and structural collapse during cycling as the repeated intercalation/deintercalation of the sizable radius of K^+^ (1.38 Å). It will easily bring on an unfavorable rate capability and rapid capacity fading with insufficient cyclic stability.^[^ ^2^, ^6^ ^]^ Therefore, although graphite has the potential to reversibly stock K^+^ between the carbon layers, and is considered the preferred anode material for PIBs, its unsatisfactory cycling life and rate performance tremendously hinder further development.^[^ ^7^ ^]^

Given the aforementioned issues, for the slow diffusion paths thanks to larger particle size and severe accumulation in graphite materials, mechanical milling is regarded as a highly practical method to reduce the particle size. As a result, the diffusion path of K^+^ can be well shortened and some defects can be introduced, thus improving the electrochemical performance of graphite.^[^ ^8^ ^]^ It is difficult, however, to directly change the inherent compact stacked structure of graphite using purely mechanical milling, so its K^+^ storage capacity and rate performance remain imperfect. Moreover, the narrow interlayer spacing in graphite materials will impede the rapid transfer/diffusion of K^+^ between carbon layers and cause a huge volume expansion. Therefore, alkali activation can be utilized to enlarge the interlayer to conform to K^+^ storage and fast movement, ameliorating the rate performance and cycle stability.^[^ ^9^ ^]^ Nevertheless, it is inevitable that some toxic and hazardous chemicals will be involved in the activation process, which should not be considered. In addition, heteroatom doping is another efficient method to enhance the electrochemical performances of graphite materials. It can incorporate a great many defects to provide effective K^+^ diffusion pathways, increase the electrochemical active adsorption sites of K^+^ storage, and promote electrical conductivity.^[^ ^10^ ^]^ Despite that, owing to the extremely high structural stability and perfect sp^2^ carbon layer of this compact graphite, it is still of big problem to realize productive heteroatom doping.^[^ ^7a^ ^]^ Although some progress has been achieved, the volume expansion during repeated cycling remains not negligible and the capacity of graphite anode is still unsatisfactory (<100 mA h g^−1^ at 1 A g^−1^), with inadequate cycling lifetime (<200 cycles).^[^ ^11^ ^]^ At the same time, it remains a challenge to balance the high capacity and initial coulombic efficiency (ICE).

The graphitization degree of a carbon material is critical to its performance. Recently, a great deal of research efforts have focused on improving the graphitization of hard carbon (HC) to obtain slight long‐range ordered graphite layers without over‐graphitization.^[^ ^12^ ^]^ The reason is that HC typically has distinct long‐range disordered and short‐range ordered graphite domains with amorphous structures and a multitude of defects and edge sites.^[^ ^13^ ^]^ The presence of abundant defects and amorphous structure inevitably causes rigorous irreversible capacity loss with minimal initial Coulombic efficiency (ICE) and low electric conductivity.^[^ ^14^ ^]^ The regulation of graphitization degree can promote the electrical conductivity of HC while minimizing superfluous irreversible ion storage sites, and provide an efficient ion/electron transport channel, thus ensuring a stable ion storage behavior.^[^ ^15^ ^]^ For instance, Zhao et al.^[^ ^16^ ^]^ utilized molecular‐level modulation to control graphitization of HC, thereby eliminating redundant defects in HC, and maintaining an effective Na^+^ ions pathway. The optimized HC with low‐defect graphitic layers showed an ultra‐high ICE of 92.05% and unsurpassed cycling performance in SIBs. Similarly, Kim et al.^[^ ^17^ ^]^ analyzed the relationship between the graphitization degree of HC and its platform capacity at low potentials in PIBs. Alvin et al.^[^ ^18^ ^]^ explored the correlation between the intercalation sites of Na^+^/K^+^ and the low‐potential‐plateau capacity of HC. However, few studies have been reported to modulate the graphitization degree of graphitic materials. In fact, regulating the graphitization degree of graphitic materials, is also very meaningful, which can significantly improve the electrochemical properties of graphitic materials, but is often easily overlooked by researchers. The reason may be that graphite materials with ultra‐high structural stability and over‐perfect sp^2^ carbon layer are not well‐regulated and lack active sites on their surfaces, which are detrimental to K^+^ storage. What's more, the volumetric capacity is indispensable for the development of miniaturization and integration of electronic products, but it has not attracted the attention of researchers in PIBs.^[^ ^19^ ^]^

Inspired by these studies, we selected highly graphitized carbon fibers (CNF) and successfully synthesized a series of nitrogen‐doped carbon‐coated carbon fiber composites with tunable graphitization (CNF@NC‐x). We utilized manganese ions to capture pyrrole monomers via in situ oxidative polymerization to form a homogeneous amorphous carbon layer on the CNF surface without the aid of any additional catalysts. Thus, abundant defects at the interface and edges could be introduced into the CNF surface as electrochemical active sites to adsorb K^+^ and as fast K^+^/electron transfer channels. More importantly, the presence of manganese ions could also catalyze the forming of short‐range curved graphite layers. The amorphous domains and short‐range curved graphite layers encapsulated on the CNF surface could provide sufficient mitigation spaces for K^+^ storage to alleviate the deleterious volume changes. The optimized CNF@NC‐5 electrode exhibited a high ICE (69.3%) and amazing reversible volumetric capacity (510.2 mA h cm^−3^ after 100 cycles at 0.1 A g^−1^) with mass capacity of 294.9 mA h g^−1^, advantageous rate performance (102.7 mA h g^−1^ at 5 A g^−1^), and long‐term cycle stability (141.9 mA h g^−1^ after 1500 cycles at 1 A g^−1^, and 98.3 mA h g^−1^ after 2000 cycles at 2 A g^−1^). The K^+^ storage mechanism and reaction kinetic analysis were studied combining in situ X‐ray diffraction (XRD) spectra, in‐situ Raman spectroscopy, in‐situ electrochemical impedance spectroscopy (EIS), and first‐principles calculation. As a result, the “1+2” model K^+^ storage mechanism of the CNF@NC‐5 electrode was verified. It involved the one‐step adsorption and two‐step insertion, i.e., surface adsorption mechanism before 0.5 V, an intercalating mechanism in the nanocrystalline graphite crystal region from 0.5 to 0.25 V, an insertion mechanism in the ordered graphite layer from 0.25 to 0.001 V. Potassium‐ion full cells and capacitors were also subsequently fabricated by assembling with a PTCDA‐450 and AC as cathode, respectively, manifesting the promising potential of practical application. This work demonstrates that it is effective and feasible to regulate the graphitization degree of graphitic materials and provides a guideline for the design of high‐performance graphite anodes.

## 2 Results and Discussion

**Figure 1:**
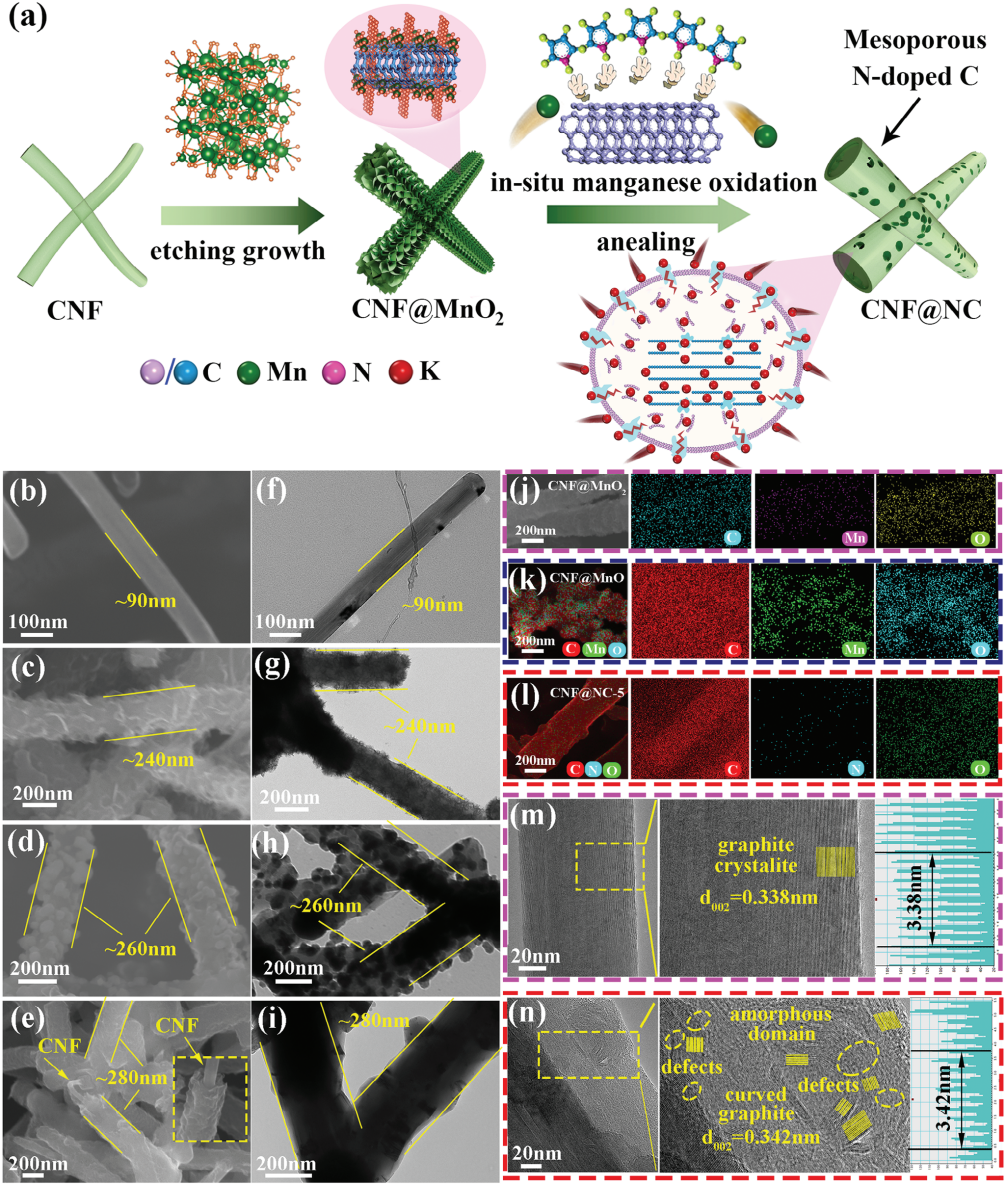
a) Schematic illustration of the synthesis process and the K^+^ storage mechanisms of the CNF@NC sample. b–i) SEM and TEM images of CNF, CNF@MnO_2_, CNF@MnO, and CNF@NC‐5 samples. j–l) The corresponding elemental mapping of CNF@MnO_2_, CNF@MnO, and CNF@NC‐5 samples. m,n) HRTEM analysis of CNF and CNF@NC‐5 samples.

The porous CNF@NC nanorods were synthesized as illustrated in **Figure** **1**a via a simple three steps process. First, CNF@MnO_2_ was synthesized through a redox reaction between KMnO_4_ and CNF, in which KMnO_4_ acted as a strong oxidizing agent and CNF acted as a reducing agent under acidic conditions (4MnO_4_^−^ + 3C + 4H^+^ → 4MnO_2_↓ + 3CO_2_↑ + 2H_2_O).^[^ ^20^ ^]^ In this process, KMnO_4_ would preferentially react with the carbon in the surface layer of CNF, after that MnO_2_ nanosheets could grow perpendicularly on the surface of CNF through an etching growth process. As shown in Figure 1b,f, the diameter of the single CNF was ca. 90 nm, while the prepared CNF@MnO_2_ was ca. 240 nm (Figure 1c,g). The EDS elemental mapping images (Figure 1j) demonstrated that C, Mn, and O elements were homogeneously distributed in CNF@MnO_2_. Afterward, CNF@NC was synthesized through an in‐situ oxidation polymerization and carbonization process. In this process, MnO_2_ played a decisive role, which acted as an excellent oxidant to trap the pyrrole monomers under acidic conditions. It could make sure that the pyrrole was uniformly coated on the CNF@MnO_2_ surface. Simultaneously, MnO_2_ would react with the acid to obtain soluble Mn^2+^ (MnO_2_ + 4H^+^ + 2e^−^→ Mn^2+^ + 2H_2_O),^[^ ^21^ ^]^ so that MnO_2_ could be removed in the subsequent cleaning process. Thus, a series of CNF@NC‐x could be obtained by regulating the amount of KMnO_4_ (x = 1, 2, 3, 4, 5, 6, and 7).

As depicted in Figure S1a,b (Supporting Information), it could be seen, when the amount of KMnO_4_ was less (x = 1 and 2), that there were some irregular blocks owing to the insufficient MnO_2_ obtained to completely capture the pyrrole monomer. Some of the residual pyrrole monomers formed irregular blocks and remained in the samples after subsequent carbonization. As the amount of KMnO_4_ increased (x = 3 and 4), these bulk gradually disappeared, although some incompletely polymerized pyrrole‐derived carbon material survived on the surface of the CNF@NC‐3 and CNF@NC‐4 for the amount of MnO_2_ obtained not optimal (Figure S1c,d, Supporting Information). This phenomenon persisted until x = 5 (CNF@NC‐5). As illustrated in Figure 1e,i, the outer layer of CNF was uniformly coated with a pyrrole‐derived carbon layer and the thickness was ≈85 nm. The existence of the pyrrole‐derived carbon layer could simultaneously serve as a conductive skeleton and a protective cushioning layer like a “battle robe” to restrain the volume variation of CNF, thus guaranteeing the structural stability of CNF. The corresponding EDS elemental mapping images (Figure 1l) exhibited that C, N, and O elements were homogeneously distributed throughout the whole sample. In contrast, when the amount of KMnO_4_ was too much (x = 6 and 7), the thickness of the encapsulated carbon layer also grew. As shown in Figure S1e,f (Supporting Information), the thickness of CNF@NC‐6 was ≈115 nm, while CNF@NC‐7 was ≈125 nm. Meanwhile, the control sample of CNF@MnO without a pyrrole‐derived carbon layer could be readily prepared via direct annealing of CNF@MnO2. As depicted in Figure 1d,h, the original MnO_2_ nanosheets on the CNF surface had completely disappeared, and the recoated particles were of different sizes. It was mainly seeing the increase in the reaction rate between MnO_2_ and CNF at high temperatures during the calcination process, resulting in the breaking of the chemical bond between CNF and MnO_2_. As a reducing agent, CNF could accept oxygen ions from MnO_2_, which led to the reduction of MnO_2_ nanosheets to MnO particles (2MnO_2_ + C → 2MnO + CO_2_↑).^[^ ^22^ ^]^ The diameter of prepared CNF@MnO was ca. 260 nm. As shown in Figure 1k, the EDS elemental mapping images unambiguously confirmed the presence of C, Mn, and O elements in CNF@MnO.

It was possible to introduce plentiful defect sites and amorphous regions on the surface of CNF through etching growth, as well as in‐situ oxidative polymerization. To verify our viewpoint, further high‐resolution TEM analyses about CNF and CNF@NC‐5 were performed. The HRTEM image of CNF (Figure 1m) demonstrated the presence of parallel lattice fringes with a highly graphitized graphite laminar structure and interplanar spacing was 0.338 nm (matching with (002) plane of graphite). Correspondingly, the HRTEM image of CNF@NC‐5 (Figure 1n) showed that the abundant defects at the interface and edges had been successfully introduced into CNF (the yellow circles). The presence of these defects could introduce plentiful active sites to adsorb K^+^.^[^ ^16^, ^21^ ^]^ At the same time, it had some amorphous domains and short‐range curved graphite layers could be observed in the magnified images, which could provide more mitigation spaces for K^+^ storage.^[^ ^1b^ ^]^ A more pronounced curved graphite layer could also be observed in Figure S2 (Supporting Information). Similarly, the interplanar spacing of 0.342 nm was attributed to the (002) plane of graphite. Thus, CNF@NC‐5 consisted of an amorphous carbon layer, a short‐range curved graphite layer, and a highly graphitized CNF. The different microstructures of the CNF@NC‐5 and CNF samples implied the graphitization degree could be successfully tuned through etching growth and in‐situ oxidative polymerization. This hierarchical multilayer structure might alleviate the volume expansion of the highly graphitized CNF during repeated K^+^ insertion/extraction and provide copious active sites to store K^+^, which was expected to obtain an outstanding rate capability and structural stability of CNF@NC‐5.

**Figure 2:**
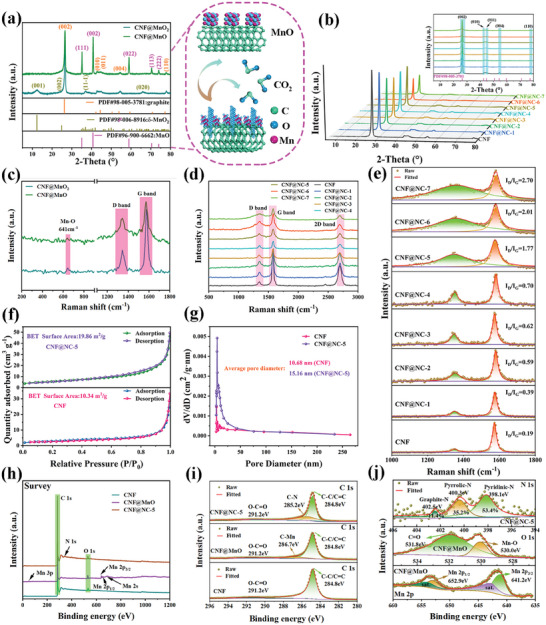
a) XRD patterns of CNF@MnO_2_ and CNF@MnO samples (Insert was the schematic diagram of the transformation of CNF@MnO_2_ into CNF@MnO). b) XRD patterns of CNF and CNF@NC‐x samples. c) Raman spectroscopy of CNF@MnO_2_ and CNF@MnO samples. d,e) Raman spectroscopy of CNF and CNF@NC‐x. f) N_2_ adsorption‐desorption isotherms, and g) the porous size distribution curves of CNF and CNF@NC‐5 samples. h) Global XPS profiles of CNF, CNF@MnO, and CNF@NC‐5 samples. High‐resolution XPS spectra of i) C 1s of CNF, CNF@MnO, and CNF@NC‐5 samples, j) Mn 2p and O 1s of CNF@MnO sample, and N 1s of CNF@NC‐5 sample.

The X‐ray diffraction (XRD) pattern (**Figure** **2**a) clearly illustrated the conversion of CNF@MnO_2_ to CNF@MnO. The diffraction peaks at 26.5°, 42.8°, 44.7°, 54.5°, and 77.8° were assigned to the (002), (010), (011), (004), and (110) planes of graphite (PDF#98‐005‐3781), respectively, and the remaining four peaks at 12.6°, 24.4°, 36.9° and 65.8° were indexed to the (001), (002), (11‐1) and (020) crystal planes of δ‐MnO_2_ (PDF#98‐006‐8916) in CNF@MnO_2_. As shown in the insert, MnO_2_ would undergo a cleavage process and react with CNF, thus releasing CO_2_ and converting to MnO at high temperatures. The diffraction peaks corresponding to MnO_2_ disappeared and those belonging to MnO appeared. The diffraction peaks at 35.1°, 40.7°, 59.0°, 70.4°, and 74.1° were attributed to crystal planes (111), (002), (022), (113), and (222) of MnO (PDF#96‐900‐6662), respectively. It was consistent with the previous discussion. Meanwhile, the presence of particularly pronounced graphite peaks in CNF@MnO_2_ and CNF@MnO samples was also indicative of the high degree of graphitization.

Figure 2b was the XRD pattern of CNF and CNF@NC‐x samples. All diffraction peaks discussed above belonging to graphite (PDF#98‐005‐3781) were found in CNF and CNF@NC‐x samples. In addition, no diffraction peaks from MnO_2_ or MnO could be detected in CNF@NC‐x samples, which could confirm that MnO_2_ had been completely converted into soluble Mn^2+^ and completely taken out during the washing process. It was also consistent with the EDS results. The peak intensity of the (002) peak would become weaker with the increase in the amount of KMnO_4_. As mentioned earlier, MnO_2_ (derived from KMnO_4_) could act as an oxidant to trap the pyrrole, ensuring that the pyrrole was encapsulated on the CNF@MnO_2_ surface. Thus, substantial interface defects and amorphous regions would be brought into the surface of CNF, which would weaken the peak intensity of the (002) peak. Raman spectra (Figure 2c) could further affirm the presence of MnO_2_, MnO, and CNF in CNF@MnO_2_ and CNF@MnO samples, respectively. The peak at 641 cm^−1^ was assigned to the Mn‐O vibration.^[^ ^23^ ^]^ And the two peaks at 1346 and 1572 cm^−1^ were attributed to the D and G bands of carbon.^[^ ^10a^ ^]^ The Raman spectra results of CNF and CNF@NC‐x samples were displayed in Figure 2d. It could be observed that the D‐band became broader, and the G‐band became weaker as the KMnO_4_ content increased. This might also be due to, as the XRD analysis, the introduction of plentiful defects and amorphous regions. The variation of the 2D‐band (≈2700 cm^−1^) could also correspond to the graphitization degree. It was well known that the D band reflected the sp^3^ carbon A_1g_ vibrations caused by defects and disordered carbon, while the G band was assigned to the symmetry and crystallinity of sp^2^ graphitized carbon.^[^ ^10a^ ^]^ Thus, the area ratio between the D and G peaks (*I*_D_/*I*_G_) could be utilized to reveal the graphitization degree of CNF@NC‐x samples. As shown in Figure 2e_,_ the *I*_D_/*I*_G_ ratio increased from 0.19 (CNF) to 2.70 (CNF@NC‐7). It again suggested that the highly graphitized CNF could be modulated by etching growth and in situ oxidative polymerization, which brought some defects and disordered regions. Similarly, as illustrated in Figure S3 (Supporting Information), it could be verified based on the variation of *L*_a_ with x‐value (from CNF to CNF@NC‐7) (L_a_ = (2.4 × 10^−10^) λ^4^ (*I*_D_/*I*_G_)^−1^, λ = 532 nm).^[^ ^24^ ^]^

Figure 2e,g presented the specific surface area and pore‐size distribution information. The N_2_ adsorption‐desorption isotherm of CNF@NC‐5 appeared to be a typical IV isotherm with H4 hysteresis loops, verifying the presence of a mesoporous structure.^[^ ^25^ ^]^ The specific surface area of CNF@NC‐5 was 19.86 m^2^ g^−1^ much larger than that of CNF (10.34 m^2^ g^−1^). Meanwhile, CNF@NC‐5 showed extensive mesopores with an average pore size of 15.16 nm and a proper pore volume (0.0764 cm^3^ g^−1^), corresponding to 10.68 nm, and 0.0515 cm^3^ g^−1^ of CNFs, respectively. It was obvious that, as detected in Figure S4 (Supporting Information), the CNF@NC‐5 had a higher contribution of mesoporous to the total surface area (57.6%) and pore volume (97.5%). The suitable mesoporous structure of CNF@NC‐5 was favorable for electrolyte diffusion and K^+^ storage, which could shorten K^+^ diffusion length and promote charge transfer.^[^ ^25^, ^26^ ^]^ Moreover, CNF@NC‐5 possessed a larger specific surface area relative to CNF, which could also provide a more efficient electrochemical reaction area for K^+^ storage, and generating an obvious pseudo‐capacitive effect, and thus boosting the electrochemical performance and charge storage efficiency.^[^ ^25^, ^27^ ^]^

The chemical compositions of samples were studied by X‐ray photoelectron spectroscopy (XPS). As shown in Figure 2h, the Mn element, corresponding to the above EDS results, was only detected in the CNF@MnO sample, which reaffirmed that MnO_2_ was completely converted into soluble Mn^2+^ and completely brought out during the washing process. Simultaneously, the C ls spectra (Figure 2f) confirmed the existence of a C‐Mn bond (286.7 eV), suggesting that the CNF@MnO was directly derived from CNF@MnO_2_. Based on the Mn 2p spectrum (Figure 2j), two characteristic peaks at 641.2 eV (Mn 2p_3/2_) and 652.9 eV (Mn 2p_1/2_) with an energy difference of 11.7 eV, and the pair of satellite peaks at 642.9 and 654.2 eV were consistent with MnO.^[^ ^28^ ^]^ The O 1s peak (Figure 2j) could be resolved into two peaks centered at 530.0 eV and 531.8 eV, attributed to the Mn‐O and C═O, respectively. This further verified that the product of direct high‐temperature treatment of CNF@MnO_2_ was CNF@MnO. Similarly, the N element could be discovered in CNF@NC‐5, and the C ls spectra (Figure 2f) proved the presence of a C─N bond (285.2 eV), which also could be verified by FTIR spectra (Figure S5, Supporting Information). The high‐resolution N 1s spectrum in Figure 2j contained graphitic‐N (402.5 eV), pyrrolic‐N (400.3 eV), and pyridinic‐N (398.1 eV). The contents of pyrrolic‐N and pyridinic‐N were as high as 88.6%, while graphite‐N was only 11.4%. Based on the XPS analysis, the N content was ≈1.5 wt.% in the whole CNF@NC‐5 sample, resulting in a non‐graphitic‐N content of nearly 1.33 wt.%. It was well known that pyridinic‐N and pyrrolic‐N, especially pyridinic‐N, could heighten the electronic conductivity of graphitic carbon and the capacitive adsorption of K^+^.^[^ ^1^, ^22^ ^]^ Therefore, the CNF@NC‐5 sample possessed a high pyrrolic‐N content (35.2%), and pyridinic‐N content (53.4%) could provide more open edge sites and defects as adsorption active sites to storage K^+^, which was favorable for enhancing the reversible capacity and improving the rate capability and the cycling lifetime.^[^ ^29^ ^]^

**Figure 3:**
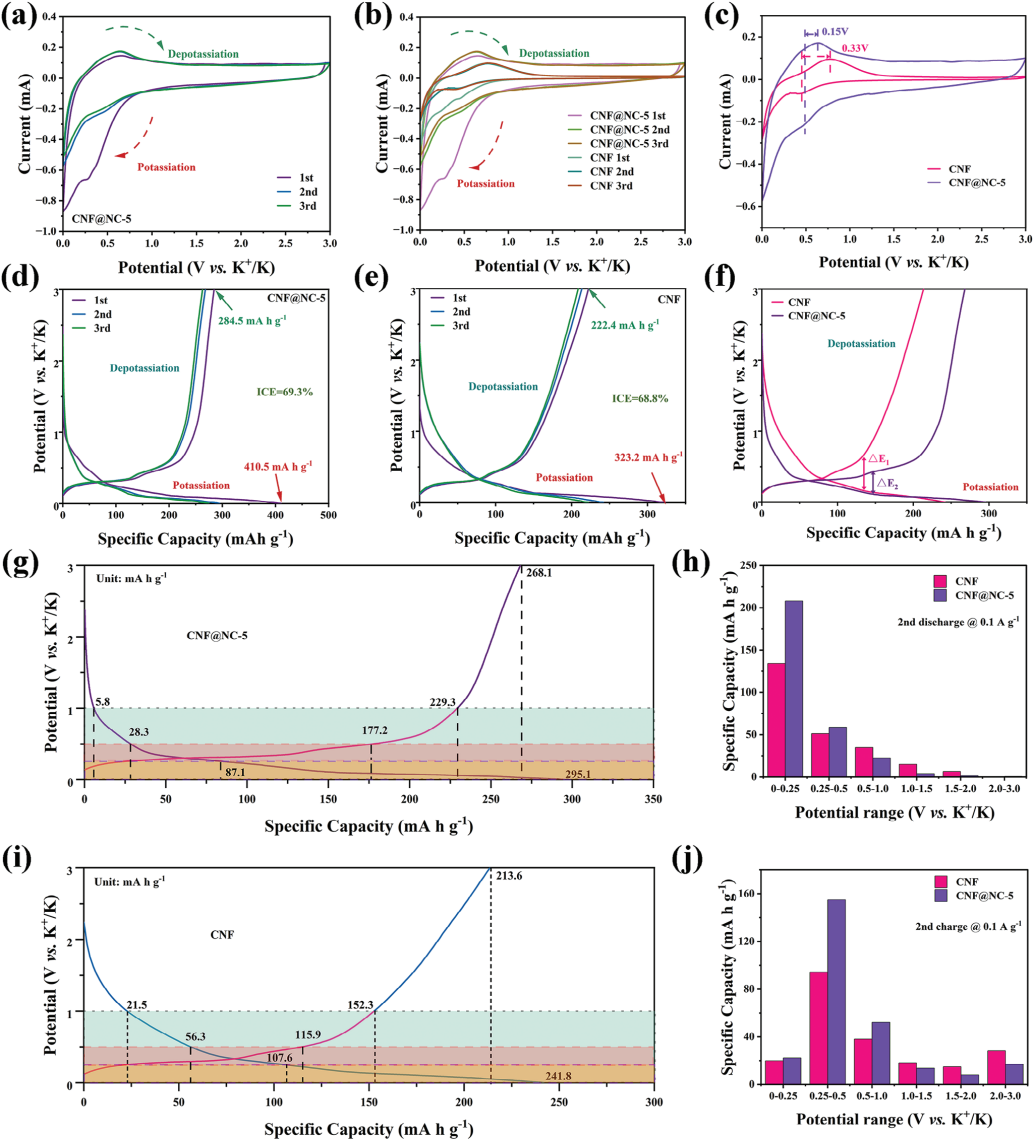
The first three CV curves between 0.001 and 3.0 V versus K^+^/K at 0.5 mV s^−1^ of a) CNF@NC‐5 electrode, and b) CNF and CNF@NC‐5 electrodes. c) The second CV curves of CNF and CNF@NC‐5 electrodes. The first three galvanostatic charge/discharge curves at 0.1 A g^−1^ of d) CNF@NC‐5 electrode, and e) CNF electrode. f) The second charge/discharge curves of CNF and CNF@NC‐5 electrodes. Schematic illustration of the second charge/discharge curves of g) CNF@NC‐5 electrode, and i) CNF electrode. Quantitative capacities of CNF and CNF@NC‐5 in different voltage zones at 0.1 A g^−1^ during h) discharging and j) charging processes.

The electrochemical performance of CNF and CNF@NC‐x samples were thoroughly characterized in 1 M KFSI in EC/DEC electrolyte to investigate the correlation between the graphitization degree of CNF and K^+^ storage behavior (CMC as a binder).^[^ ^30^ ^]^ In the first cathodic scan, the CNF@NC‐5 electrode (**Figure** **3**a) exhibited a broader peak appeared at 0.2–0.6 V and then disappeared during the subsequent scans, corresponding to the decomposition of electrolyte and the formation of the SEI layer.^[^ ^31^ ^]^ The well overlapping in the subsequent 2nd and 3rd scans indicated that the CNF@NC‐5 electrode had good electrochemical reversibility. There was a positive correlation between the area of the CV curve and the capacity of the material.^[^ ^25^ ^]^ As could be viewed in Figure 3b, the CV curve of the CNF@NC‐5 electrode had a larger area, implying that CNF@NC‐5 had a higher specific capacity than CNF, which could also be known from their charging and discharging curves. As discovered in Figure 3d,e, initial discharge and charge specific capacities of CNF@NC‐5 were 410.5 and 284.5 mA h g^−1^, respectively, which were higher than those of CNF at 323.2 and 222.4 mA h g^−1^, and CNF@NC‐5 showed a higher initial coulombic efficiency (ICE) of 69.3%. Meanwhile, CNF@NC‐5 possessed a smaller polarization (0.15 V versus 0.33 V for CNF) and voltage hysteresis (Δ*E*_2_ < Δ*E*_1_) as observed in Figure 3c,f. Low polarization was critical for batteries in practical applications and the energy efficiency would be affected by voltage hysteresis.^[^ ^13^ ^]^ Therefore, the capacity, ICE, and electronic conductivity of the material could be ameliorated to some extent. In addition, the first charge‐discharge curves of CNF@NC‐x samples (x = 1, 2, 3, 4, 6, and 7) were shown in Figure S6 (Supporting Information), and the corresponding initial discharge/charge specific capacities and ICE were given in the table. It demonstrated that the modulation of the graphitization degree of CNF could improve its electrochemical properties by introducing defects and an amorphous carbon layer on the highly graphitized CNF surface. It also suggested that a high ICE and capacity could be balanced through precise modulation and continuous optimization, without causing excessive irreversible capacity loss.

The charge/discharge data was obtained through LAND‐CT2001A, so the volumetric capacity could be calculated using the following equation:^[^ ^19^, ^32^ ^]^

(1) $$C_{V}\;\left(\mathrm{mA}\;h\;cm^{-3}\right)=C_{g}\;\left(\mathrm{mA}\;h\;g^{-1}\right)\times \rho \left(g\;cm^{-3}\right)$$

(2) $$\rho =\frac{1}{V_{\mathrm{total}}+1/\rho_{\mathrm{carbon}}}$$

In this formula, *C*_v_ was volumetric capacity of the electrode (mA h cm^−3^), *C*_g_ was specific gravimetric capacity of the electrode (mA h g^−1^), ρ was density of material (g cm^−3^), *V*_total_ was the pore volume from N_2_ adsorption‐desorption isotherm (cm^3^ g^−1^), and *ρ*_carbon_ was the true density of carbon (2 g cm^−3^). According to the results of the N_2_ isotherm, the density of CNF was 1.81 cm^3^ g^−1^, and CNF@NC‐5 was 1.73 cm^3^ g^−1^. Thus, the CNF@NC‐5 electrode also exhibited a higher reversible volumetric capacity of 492.2 mA h cm^−3^ than CNF (402.5 mA h cm^−3^).

For the CNF@NC‐5 electrode exhibiting a superior capacity than CNF, a detailed analysis of the capacity contribution was conducted. In the dQ/dV curves of the CNF@NC‐5 electrode (Figure S7, Supporting Information), three reduction peaks (0.50 V, 0.25 V, and 0.10 V) and two oxidation peaks (0.32 V and 0.46 V) could be monitored, which might correspond to the step transformation processes of K^+^ storage. Based on the results of the above analysis, the second charge/discharge curves of CNF@NC‐5 (Figure 3g) and CNF (Figure 3i) electrodes could be divided into different stages and compared to the capacity of each region. As shown in Figure 3h, the CNF@NC‐5 displayed a large proportion of the discharge capacity below 1.0 V (98.0% vs. 91.1% of CNF) with considerable capacities of 289.3 mA h g^−1^. Interestingly, CNF@NC‐5 had the ability to provide a higher capacity below 0.25 V compared to CNF (208 mA h g^−1^ vs. 134.2 mA h g^−1^). Meanwhile, the charge capacity (Figure 3j) of the CNF@NC‐5 electrode below 1.00 V accounted for 85.5% with noticeable capacities of 229.3 mA h g^−1^, while CNF was 71.3% and 152.3 mA h g^−1^, respectively. Correspondingly, CNF@NC‐5 was capable of providing a capacity of 177.2 mA h g^−1^ below 0.5 V compared to CNF (114.2 mA h g^−1^). The results revealed that the introduction of defects and disordered regions on the CNF surface by manipulating graphitization could conspicuously promote the capacity at low potentials.

**Figure 4:**
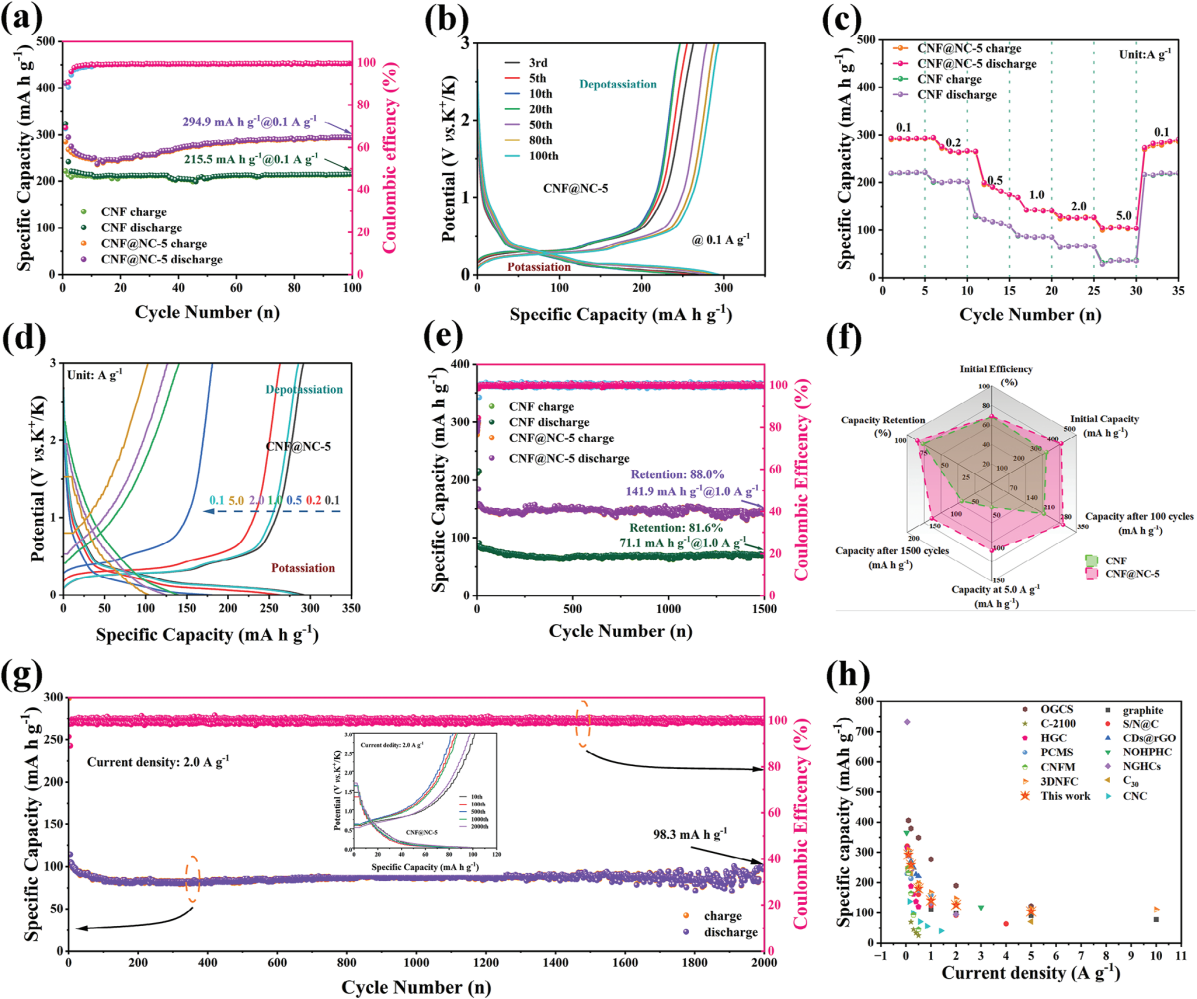
a) Potassiation and depotassiation capacity and coulombic efficiency of CNF and CNF@NC‐5 electrodes at 0.1 A g^−1^. b) The charge‐discharge curves for selected cycles of CNF@NC‐5 electrode at 0.1 A g^−1^. c) The rate capability of CNF and CNF@NC‐5 electrodes from 0.1 to 5.0 A g^−1^. d) The charge‐discharge curves at various current densities of CNF@NC‐5 electrode (0.1, 0.2, 0.5, 1.0, 2.0, 5.0, and 0.1 A g^−1^). e) Potassiation and depotassiation capacity of CNF and CNF@NC‐5 electrodes at 1.0 A g^−1^. f) Comparison of all aspects of CNF and CNF@NC‐5 electrodes. g) Long‐term cycle stability and coulombic efficiency at a high current density of 2.0 A g^−1^ of CNF@NC‐5 electrode. h) Comparison of rate capability of CNF@NC‐5 with other reported carbon materials in PIBs.

To further clarify that the modulation of the graphitization degree had a significant effect on the electrochemical properties of the material, a more comprehensive analysis was performed. The cycling performance of CNF and CNF@NC‐5 electrodes over 100 cycles at 0.1 A g^−1^ were depicted in **Figure** **4**a. CNF@NC‐5 electrode had a higher specific capacity and maintained at 294.9 mA h g^−1^ after 100 cycles than CNF (215.5 mA h g^−1^). While maintaining a high mass‐specific capacity, the CNF@NC‐5 electrode also had an extremely high volumetric capacity (510.2 mA h cm^−3^) than CNF (390.1 mA h cm^−3^). The high volumetric capacity could be ascribed to the fact that CNF@NC‐5 had a high density. It was since that the CNF@NC‐5 prepared by etching growth and in situ oxidative polymerization, despite the existence of a disordered carbon layer on the CNF surface and the introduction of some defects, had not an ultra‐high specific surface area as most of the porous carbon materials reported so far.^[^ ^1^, ^13^, ^26^, ^33^ ^]^ CNF@NC‐5 still maintained a relatively low specific surface area (only 19.86 m^2^ g^−1^), even though it was higher than that of CNF (10.34 m^2^ g^−1^). Therefore, the CNF@NC‐5 electrode had a fantastic volumetric capacity as compared to literature reports,^[^ ^19^, ^33^, ^34^ ^]^ which was slightly lower than the theoretical volumetric capacity (Table S1, Supporting Information).^[^ ^35^ ^]^ The capacity of two electrodes slowly increased during the cycle. The bizarre capacity increase might be attributed to the forming of a stable SEI film, as well as the slow infiltration of electrolytes, which promoted electrode activation and equipped increasingly active materials to participate in electrochemical reactions.^[^ ^36^ ^]^ The charge–discharge curves for selected cycles of CNF@NC‐5 and CNF electrode at 0.1 A g^−1^ could also verified it. As displayed in Figure 4b and Figure S8 (Supporting Information), the 100th charge/discharge capacity was significantly higher than the other cycles.

As expected, the CNF@NC‐5 electrode exhibited an excellent rate performance (Figure 4c). The reversible capacities of the CNF@NC‐5 were 291.7 (4th), 263.2 (9th), 181.2 (14th), 141.1 (19th), 127.0 (24th), and 102.7 (29th) mA h g^−1^, at 0.1, 0.2, 0.5, 1.0, 2.0, and 5.0 A g^−1^, respectively. As the current density turned back to 0.1 A g^−1^, the reversible capacity was slightly reduced (285.6 mA h g^−1^, 34th). It was also superior to CNF with the reversible capacities of 221.4 (4th), 202.1 (9th), 114.4 (14th), 85.8 (19th), 66.7 (24th), 36.1 (29th), and 215.8 (34th) mA h g^−1^ at current densities of 0.1, 0.2, 0.5, 1.0, 2.0, 5.0 and turned back to 0.1 A g^−1^, respectively. The galvanostatic charge/discharge profiles for selected cycles (Figure 4d; Figure S9, Supporting Information) well coincided with each other. Remarkably, when the current density was raised to 1.0 A g^−1^ after active 10 cycles at 0.1 A g^−1^, as shown in Figure 4e and Figure S10 (Supporting Information), over 1500 cycles, the CNF@NC‐5 electrode could maintain 141.9 mA h g^−1^. The CE closed to 100% and the capacity retention rate was 88.0% with a capacity fading of 0.0147% per cycle (corresponding to the 10th cycle), which demonstrated the exceptional cycle stability of the CNF@NC‐5 electrode. In comparison, the CNF electrode (Figure 4e; Figure S11, Supporting Information), delivered inferior reversible capacities of 71.1 mA h g^−1^ after 1500 cycles and the capacity retention was 81.6%. In addition, a full comparison of CNF and CNF@NC‐5 electrodes was represented in Figure 4f, and the electrochemical performance of CNF@NC‐x (x = 1, 2, 3, 4, 6, and 7) electrodes and comparison were displayed in Figures S12–S18 (Supporting Information). It also further manifested that the electrochemical properties of CNF could be improved by regulating the graphitization degree of CNF to introduce defects and amorphous carbon layer on the highly graphitic CNF surface. The CNF@NC‐5 sample with a high N content and specific surface area could provide more open edge sites and defects to storage K^+^, which was conducive to enhancing the reversible capacity and strengthening the rate capability and the cycle life without causing excessive irreversible capacity loss.

Furthermore, the long cycling stability was also investigated at 2.0 A g^−1^ (Figure 4g). After 2000 cycles, a reversible capacity of 98.3 mA h g^−1^ and the CE value close to 100% could be still obtained, which suggested that the CNF@NC‐5 electrode had terrific cycling stability in KIBs. The SEM images of the CNF@NC‐5 after 2000 cycles could be obtained in Figure S19 (Supporting Information). It could be noted that even after 2000 cycles, the electrodes remained intact without peeling or cracking, and the CNF@NC‐5 basically maintained its original morphology without significant changes. The size of the single fibers before and after cycling had a negligible change (from 280 to 285 nm). The results illustrated that the volume expansion of the material was well suppressed and the superior structural stability of CNF@NC‐5. The reason could be ascribed to the existence of an amorphous domain carbon layer as a cushioning protective layer, which ensured the structural stability of CNF@NC‐5. The EDS elemental mapping images showed that C and N elements were distributed throughout the CNF@NC‐5. Meanwhile, innumerable O, S, and K elements were distributed in the periphery of C and N. The K and S elements were mainly from the electrolyte, which proved that an SEI layer was formed and encapsulated in the periphery of the CNF@NC‐5. It ascertained that the CNF@NC‐5 surface was coated with a stable SEI layer containing K and S elements, which ensured the structural stability of CNF@NC‐5 during cycling.^[^ ^25^, ^37^ ^]^ The electrochemical performance of CNF@NC‐5 was superior to most of the reported carbon materials (Figure 4g; Table S2, Supporting Information),^[^ ^14^, ^19^, ^29^, ^38^ ^]^ reflecting the superiority of CNF@NC‐5 as PIBs anode material.

**Figure 5:**
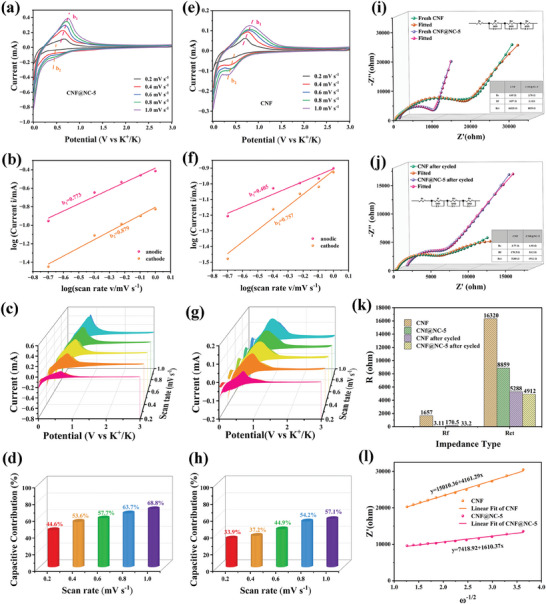
Quantitative capacitive analysis of K‐ion storage behavior: CV curves at different sweep rates of a) CNF@NC‐5 electrode, and e) CNF electrode. Fitted plots of log (i) and log (v) of oxidation and reduction peaks of b) CNF@NC‐5 electrode, and f) CNF electrode. Capacitive contribution at 0.2–1.0 mV s^−1^ of c) CNF@NC‐5 electrode, and g) CNF electrode. Normalized contribution ratio of the capacitive and diffusion‐controlled charge at different scan rates of d) CNF@NC‐5 electrode, and h) CNF electrode. EIS curve of CNF and CNF@NC‐5, and the corresponding equivalent circuit (inset): i) fresh and j) after 1500 cycles. k) Comparison of the *R*_f_ and *R*_ct_ of CNF and CNF@NC‐5 before and after cycling. l) Z’(Ω) and ω^−1/2^ curves of the CNF and CNF@NC‐5 electrodes in the low frequency range.

Given the superior electrochemical performance of the CNF@NC‐5 electrode, the K^+^ storage behaviors were investigated by CV at various scan rates of 0.2 to 1 mV s^−1^ (**Figure** **5**a). The CV curves, as the increase of scan rate, displayed similar shapes with broadened characteristic peaks, suggesting low polarization and the pseudo‐capacitive characteristic.^[^ ^39^ ^]^ Similar CV curves of CNF electrode were shown in Figure 5e. The capacitive contribution could be qualitatively evaluated based on the following equation:

(3) $$i=av^{b}$$

where *i*, *ν*, *a*, and *b* represented the peak current, scan rate, and adjustable parameters, respectively.^[^ ^39^ ^]^ And the b‐value could be obtained by the slope of the linear plot of log(|i|) versus log(ν).^[^ ^40^ ^]^

The CNF@NC‐5 electrode (Figure 5b) showed b‐values of two current peaks were 0.773 (b_1_) and 0.879 (b_2_), respectively, representing the combination of the capacitive behavior and diffusion‐controlled process in the reversible K^+^ de‐/intercalation process.^[^ ^10a^ ^]^ In comparison, for the CNF electrode, b‐values of two current peaks were 0.405 (b_1_) and 0.757 (b_2_), respectively (Figure 5f), suggesting that the diffusion‐controlled intercalation process played a predominantly role in the K^+^ storage process at the CNF electrode. Further quantitative analysis was based on the following equation:

(4) $$i\;\left(V\right)=k_{1}\;v+k_{2}v^{1/2}$$

The response current i corresponded to a fixed voltage(V), and was deemed as a combination of the surface capacitive controlled process (k_1_ν) and diffusion controlled process (*k*_2_ *ν* ^1/2^).^[^ ^41^ ^]^

Figure 5c,g exhibited the area ratio of the capacitive contribution (CNF@NC‐5 and CNF electrodes) to the whole capacitance for different scan rates. The results were illustrated in Figure 5d,g, corresponding to sweep speeds 0.2, 0.4, 0.6, 0.8, and 1.0 mV s^−1^, respectively. As depicted in Figure 5d, the proportion of the pseudocapacitive contribution of CNF@NC‐5 electrode gradually increased from 44.6% to 68.8% with the increasing scan rate. Correspondingly, the CNF electrode (Figure 5h) presented a low pseudocapacitive contribution proportion from 33.89% to 57.1%, respectively. It revealed an outstanding kinetic behavior that could further explain the exceptional electrochemical performance of the CNF@NC‐5 electrode.

The electrochemical impedance spectra (EIS) of CNF and CNF@NC‐5 electrodes before and after 1500 cycles were fitted with an equivalent circuit (Figure S20, Supporting Information) to illustrate the kinetic characteristics, and the fitted impedance data were summarized in Figure 5i,j. The obtained Nyquist plots of CNF and CNF@NC‐5 electrodes were similar in shape before and after cycling, and consisted of a charge transfer process (the high‐frequency region), and the diffusion of K^+^ (the low‐frequency region). According to the fitting results, the CNF@NC‐5 electrode possessed superior diffusion kinetics and lower charge‐transfer resistance than the CNF electrode both before and after cycling. It reaffirmed that modulating the graphitization degree of CNF, by the introduction of defects and amorphous domains, could ameliorate the charge‐transfer kinetics and kinetically favored the rapid transport of K^+^ and electrons during the electrochemical process, resulting in the superior rate capability of K^+^ storage. In comparison, after 1500 cycles (Figure 5k), the *R*_f_ and *R*_ct_ of both CNF and CNF@NC‐5 electrodes were smaller than those of fresh ones, manifesting the existence of an active process. It also helped to optimize cycling and rate performance. The diffusion coefficient of K^+^ could be calculated by the following formula: (the corresponding paraments could be found in Supporting Information)

(5)

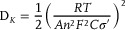




The calculated diffusion coefficients of K^+^ for CNF@NC‐5 and CNF electrodes (Figure 5l) were 7.31 × 10^−8^ and 2.83 × 10^−8^ cm^2^ s^−1^, respectively. It was further confirmed that regulating the graphitization degree of CNF, by introducing defects and amorphous domains, facilitated the diffusion of K^+^, which could clarify the fantastic capacity retention and terrific rate capability of the CNF@NC‐5 electrode during the cycling.

**Figure 6:**
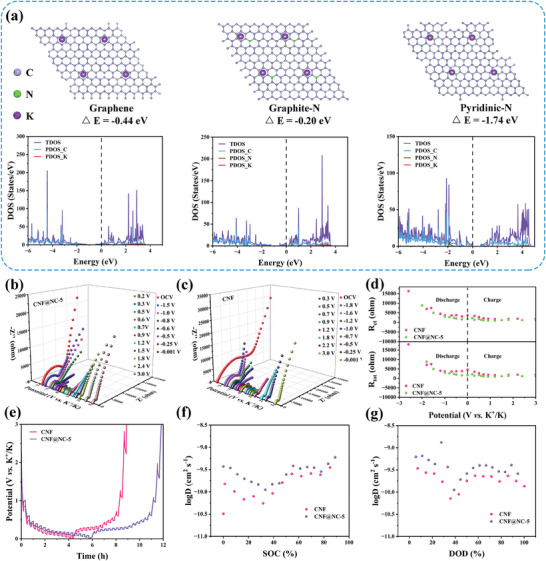
a) Top views of K atom absorbed in graphene (undoped), graphite‐N doped carbon, and pyridinic‐N doped carbon with corresponding K^+^ adsorption energies and density of states (DOS). b) and c) In situ EIS Nyquist plots of CNF@NC‐5 and CNF electrodes at preselected potentials during the initial cycle at 0.001–3.0 V, and d) the corresponding *R*_ct_ and *R*_tot_ (*R*_tot_ = *R*_s_ + *R*_f_ + *R*_ct_). e) GITT potential profiles, and f,g) the corresponding K^+^ diffusion coefficient of CNF@NC‐5 and CNF electrodes in SOC and DOD states.

It was well known that the presence of pyridinic‐N was usually surrounded by defects which could efficiently increase the number of active sites on the carbon skeleton for K ion storage, and boost the electronic conductivity of graphitic carbon.^[^ ^7a^ ^]^ It was favorable for enhancing the reversible capacity, rate capability, and cycling lifetime.^[^ ^29^, ^42^ ^]^ As shown in **Figure** **6**a, the adsorption energy of pyridinic‐N doped carbon for K atom was greatly improved (Δ*E*_a_ = −1.74 eV) compared to graphene (Δ*E*_a_ = −0.44 eV) and graphite‐N doped carbon (Δ*E*_a_ = −0.20 eV). The unveiled that CNF@NC‐5 was more favorable for the adsorption of K atoms, thereby increasing the K^+^ storage capacity of adsorption‐controlled,^[^ ^10a^ ^]^ which was consistent with the pseudo‐capacitance analysis. What's more, the corresponding density of states (DOS) analysis also revealed significant enhancement near the Fermi energy level for CNF@NC‐5 after the absorption of K atoms. It suggested that there was a strong interaction between K atoms and CNF@NC‐5, which could promote the transfer of electrons from the K atoms to the CNF@NC‐5 structure with a higher electronic conductivity. As shown in Figure S21 (Supporting Information), the CNF@NC‐5 electrode had a smaller activation energy (*E*_a_) of 8.99 kJ mol^−1^ than CNF (14.31 kJ mol^−1^) based on the EIS measurement at different temperatures (i.e., 40, 45, 50, 55, and 60 °C), which further demonstrated that the K^+^ diffusion barriers of CNF@NC‐5 were much lower. All of these benefits contributed to the dynamic kinetics, superior rate capability, and stable capability of CNF@NC‐5 electrode for PIBs.

The in‐situ EIS analysis of the CNF@NC‐5 electrode was also conducted to analyze the reaction dynamics during the first cycle (Figure 6b). Figure 6d showed the variation in the *R*_ct_ and *R*_tot_ during the initial potassiation/depotassiation process. The analysis was based on the above dQ/dV curve results (Figure S7, Supporting Information). During the discharge process, the *R*_ct_ and *R*_tot_ values decreased significantly from the open‐circuit voltage (OCV) state to 0.5 V as K^+^ enriched on the surface of the CNF@NC‐5 electrode and adsorbed on the defects, indicating that a conductive SEI film was formed at this stage.^[^ ^33d^ ^]^ Then, with the depth of discharge increased (discharging to 0.25 V), more and more K^+^ filled the defect sites and gradually embedded in the short‐range curved graphite layers, which led to the *R*_ct_ and *R*_tot_ values reduced continually. When the deep discharge continued to 0.001 V, the *R*_ct_ and *R*_tot_ values would increase slightly between 0.25 V and 0.001 V. It was associated with the continuous K^+^ insertion from the nanocrystalline graphite crystal region into the long‐ordered graphite layer, where the diffusion barrier would increase. A similar phenomenon could be observed in the CNF electrodes (Figure 6c,d) and occurred earlier. After 1.0 V, the *R*_ct_ and *R*_tot_ values began to trend upward and reached a maximum at 0.001 V, which could be attributed to the one‐step insertion mechanism of the CNF electrode. In contrast, during the subsequent charging process, the *R*_ct_ and *R*_tot_ values first decreased sharply, then increased slightly, and finally remained essentially unchanged, suggesting that the charge transfer and reaction kinetics were much faster during the depotassiation process.^[^ ^26^ ^]^ Notably, the *R*_ct_ and *R*_tot_ values of the CNF@NC‐5 electrode were lower than those of the CNF throughout the charging and discharging process, which further manifested that the CNF@NC‐5 electrode had a faster kinetic performance.

To validate the above results, Galvanostatic intermittent titration technique (GITT) measurements also were performed (Figure 6e). According to Fick's second law, when voltage was linearly proportional to ∆t^1/2^, D_k_ could be obtained by the following equation: (the corresponding determination in Figure S22, Supporting Information).^[^ ^43^ ^]^

(6) $$D_{k}=\frac{4}{\pi \Delta t}\;{\left(\frac{n_{B}V_{M}}{S}\right)}^{2}{\left(\frac{\Delta E_{s}}{\Delta E_{t}}\right)}^{2}$$

Overall, the diffusion coefficient of the CNF@NC‐5 electrode (10^−10.0^–10^−8.75^) was always higher than those of the CNF electrode (10^−10.5^–10^−9.5^) during cycling, suggesting faster K^+^ diffusion kinetics (Figure 6f,g), which could be further understood the unsurpassed rate capability of CN@NC‐5 electrode. Consistent with the description of in situ EIS, the K^+^ would preferentially enrich on the surface of the CNF@NC‐5 electrode and adsorbed on the active sites until the discharge reached 40%. As more and more K^+^ were adsorbed onto the surface active sites, a stronger and stronger repulsive force developed between them, thus resulting in a gradual decrease in the K^+^ diffusion coefficient.^[^ ^12b^ ^]^ As the discharge proceeded, the diffusion coefficient of the CNF@NC‐5 electrode showed the first stage of increase. It was due to the progressive embedding of a large amount of K^+^ from the electrode surface into the graphite nanocrystalline layer (short‐range curved graphite layers), while the number of K^+^ enriched in the periphery decreased accordingly, and the mutual repulsion force was subsequently weakened. When the first growth phase ended and entered the second phase, the diffusion coefficient of the CNF@NC‐5 electrode could possess a sharp upward pull, which could be attributed to the continuous K^+^ insertion from the nanocrystalline graphite crystal region into the long‐ordered graphite layer. The corresponding two phases could also be represented in the charging process. The above results reaffirmed the K^+^ storage mechanism of the CNF@NC‐5 electrode was in the “1+2” model. It involved the one‐step adsorption storage and two‐step insertion, i.e., surface adsorption mechanism before 0.5 V, an intercalating mechanism in the nanocrystalline graphite crystal region from 0.5 to 0.25 V, an insertion mechanism in the ordered graphite layer from 0.25 to 0.001 V. The “1+2” model was also helpful in ensuring the structural stability of CNF@NC‐5, avoiding the drastic structural expansion brought about by the sudden embedding/de‐embedding of plenteous K^+^. Adsorption followed by stepwise insertions could avoid abrupt structural changes, thus guaranteeing the cycling stability of the CNF@NC‐5 electrode.

**Figure 7:**
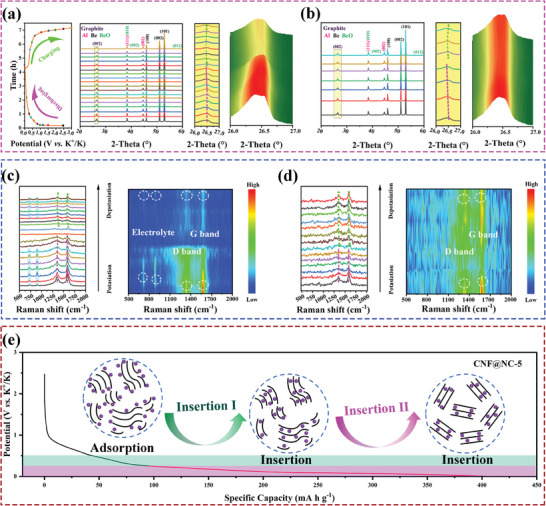
a) and b) The charge‐discharge curve at 0.1 A g^−1^ for the initial cycle and the second cycle, respectively (different potential states are marked with different colors) and corresponding in situ XRD patterns of the CNF@NC‐5 electrode. c) and d) In situ Raman patterns and corresponding contour map of CNF@NC‐5 electrode at various discharged/charged states during the initial and second charge‐discharge process, respectively. e) Schematic diagram of electrochemical energy storage mechanism of CNF@NC‐5 electrode in PIBs.

Likewise, in‐situ XRD and in‐situ Raman tests also were utilized to investigate the structural evolution and K^+^ storage mechanism of the CNF@NC‐5 electrode during potassiation/depotassiation. As shown in **Figure** **7**a, in the first stage of the discharge process (>0.5 V), the corresponding (002) peak was not significantly shifted, and the intensity of the diffraction peak was only slightly weakened for the reason that K^+^ enriched and adsorbed on the CNF@NC‐5 surface without intercalated into the graphite layer. It would not lead to significant structural changes. When discharged sequentially to 0.25 V and 0.001 V, the intensity of (002) would continue to weaken and slightly shift to a lower angle, which was equivalent to the successive intercalation of K^+^ into the graphite layer. It could be inferred that the structural change was negligible.^[^ ^44^ ^]^ During the subsequent reverse charging process, the (002) peak gradually strengthened and recovered to its original position owing to the reversibly de‐insertion of K^+^. The intensity changes of (002) peaks could be more visually illustrated in the 3D XRD patterns. Similarly, the in situ XRD spectra of the second cycle (Figure 7b) could reflect a comparable variation, which could be concluded that the structural transformation of CNF@NC‐5 was reversible.

The reversible structure transformation was also identified by further in situ Raman tests. It was in line with the description of in‐situ XRD. As displayed in Figure 7c, in the first stage, the positions of the D‐ and G‐band peaks stayed the same, but the intensity of the peaks was weakened, and the D‐band peak was gradually broadened, demonstrating that K^+^ was mainly adsorbed on the CNF@NC‐5 surface at this stage. As discharge deepened, the D‐ and G‐band peaks would mildly redshift with a weakened signal, which might be due to the insertion of K^+^ in the graphite layer. In the following charge process, the D‐ and G‐band peaks would reversibly blueshift and gradually recover to the pristine state. In the color maps, the variation trend of the D‐ and G‐band was more pronounced. Similarly, the in situ Raman spectra of the second cycle (Figure 7d) could reflect similar changes, which precisely determined that CNF@NC‐5 underwent a reversible structural change. Interestingly, two peaks belonging to the electrolyte were detected in the in situ Raman test of the first cycle, located at 600–750 and 850–1000 cm^−1^, respectively. The intensity of the two peaks gradually increased and then weakened and eventually diminished during the discharge process, and slowly reappeared when the charging state was reached, which corresponded to the generation of the SEI layer on the CNF@NC‐5 surface. Notably, the phenomenon was not observed in the in situ Raman test of the subsequent cycle, which suggested that a stable SEI film had been formed during the initial cycle, which was conducive to ensuring the stability of the CNF@NC‐5 electrode. It was consistent with the CV results. Therefore, the K^+^ storage mechanism in the CNF@NC‐5 electrode could be shown in Figure 7e, i.e., the adsorption‐insertion‐insertion mechanism.

**Figure 8:**
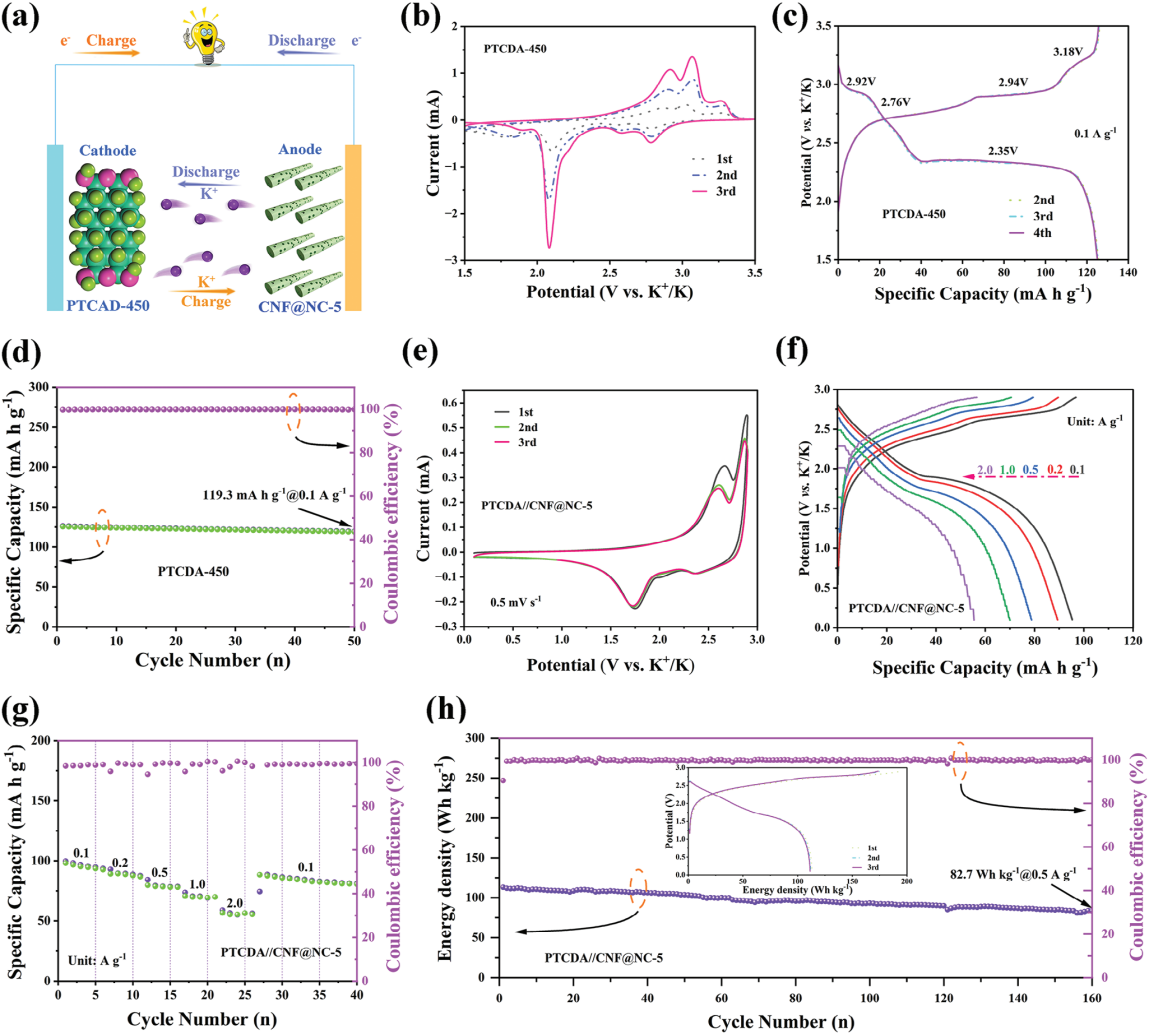
a) Schematic illustration of the PTCDA/CNF@NC‐5 PIBs. PTCDA cathode: b) CV curves between 1.5 and 3.5 V versus K^+^/K at 0.5 mV s^−1^; c) the 2nd, 3rd, and 4th charge‐discharge curves at 0.1 A g^−1^; d) potassiation and depotassiation capacity and coulombic efficiency at 0.1 A g^−1^. PTCDA//CNF@NC‐5 PIBs: e) CV curves between 0.1 and 2.9 V versus K^+^/K at 0.5 mV s^−1^; f) the charge–discharge curves at various current densities (0.1, 0.2, 0.5, 1.0, and 2.0 A g^−1^); g) the rate capability from 0.1 to 2.0 A g^−1^; h) Cycling performance of the full‐cell at 0.5 A g^−1^ over 160 cycles.

The practical application of the CNF@NC‐5 electrode was assessed by assembling the PTCDA//CNF@NC‐5 K‐ion full cells (PTCDA‐450 as cathode and CNF@NC‐5 as the anode, **Figure** **8**a). The electrochemical properties of the PTCDA‐450 electrode were depicted in the Figure 8b–d at 1.5–3.5 V. To better match the capacity of the two electrodes, the mass ratio of the PTCDA/CNF@NC‐5 PIBs was 2:1 (cathode: anode). The first three CV curves of PTCDA//CNF@NC‐5 PIBs was shown in Figure 8e at 0.1–2.9 V. The PTCDA//CNF@NC‐5 PIBs exhibited a splendid rate performance (Figure 8f and g) (based on anode and cathode mass loading). With increasing current density, the full cells delivered a capacity of 96.8, 89.6, 79.4, 70.5, and 56.6 mA h g^−1^ at 0.1, 0.2, 0.5, 1.0, and 2.0 A g^−1^, respectively. Remarkably, the energy density could be maintained at 82.7 Wh Kg^−1^ after 160 cycles at 0.5 A g^−1^, which demonstrated that the CNF@NC‐5 had some value in practical PIBs.

Again, the potassium ion hybrid capacitors (PIHCs) with CNF@NC‐5 anode and activated carbon (AC) cathode (CNF@NC‐5/AC PIHCs) were also prepared to evaluate the practicability of CNF@NC‐5 electrode (Figure S23a, Supporting Information). During the charging and discharging, the CNF@NC‐5 anode could insert/remove K^+^, and the AC cathode could adsorb/desorb FSI^−^. The electrochemical properties of AC electrode were represented in Figure S23b–d (Supporting Information) at 2.0–1.0 V. Encouragingly, the CNF@NC‐5//AC PIHCs (Figure S23e, Supporting Information) displayed an extremely long cycle life, with an energy density of 68.5 Wh kg^−1^ and retention rate of 75.9% after 3000 cycles at 1.0 A g^−1^ at 0.01–4.0 V.

## 3 Conclusions

In summary, we designed and successfully synthesized a series of nitrogen‐doped carbon‐coated carbon fiber composites with tunable graphitization (CNF@NC‐x) through etching growth, in situ oxidative polymerization, and subsequent carbonization process. The material characterization tests, and electrochemical property evaluations indicated that it was feasible to modulate the graphitization degree of CNF using an in situ chemical method. Thus, the problem of the lack of active sites on the surface of highly graphitized CNF, which was detrimental to the storage of K^+^, could be solved. In addition, we proposed and verified a “1+2” model K^+^ storage mechanism in the CNF@NC‐5 electrode. At the same time, we detected the formation process of solid electrolyte interphase (SEI) film during the initial charging and discharging using in situ Raman spectroscopy, which was invaluable for understanding the electrochemical reaction process of PIBs. Potassium‐ion full cells and capacitors were also subsequently fabricated by assembling with a PTCDA‐450 and AC as cathode, respectively, manifesting the promising potential of practical application. This work demonstrates that it is effective and feasible to regulate the graphitization degree of graphitic materials and provides a guideline for the design of high‐performance graphite anodes.

## 4 Experimental Section

### Synthesis of CNF@MnO_2_

CNF@MnO_2_ was synthesized through a redox reaction, in which KMnO_4_ acted as a strong oxidizing agent and CNF acted as a reducing agent under acidic conditions. Typically, 0.1 g CNF (XFNANO) was uniformly dispersed into 100 mL deionized water (DI water) under ultrasound for 2 h. Subsequently, 1.0 g KMnO_4_ was dissolved into the above solution by vigorous stirring for 2 h under 60 °C. Next, 0.5 mL H_2_SO_4_ (95%−98%) was added to the above solution. After stirring continuously for 2 h, the product was collected by centrifugation washed several times with DI water and ethanol, and dried at 70 °C overnight.

### Synthesis of CNF@NC

CNF@NC was synthesized through an in situ oxidation polymerization and carbonization process. Specifically, the obtained CNF@MnO_2_ (0.2 g) was dissolved in 60 mL 0.1 M HCl solution by ultrasonic treatment. Afterward, 150 µL of pyrrole monomer (Aldrich) was slowly added dropwise into the solution and stirred at 30 °C for 6 h. The formed precipitate was vacuum filtered and washed with DI water/ethanol several times until the filtrate was colorless and neutral, and subsequently dried at 70 °C overnight. Finally, the black product was annealed at 800 °C for 2 h in N_2_ with a ramp rate of 2 °C min^−1^ to obtain CNF@NC (CNF@NC‐5). To optimize the amount of MnO_2_, different amounts of KMnO_4_ (0.2, 0.4, 0.6, 0.8, 1.2, and 1.4 g) were used in the above synthesis process of CNF@MnO_2_, and the corresponding products after the synthesis of CNF@NC were denoted as CNF@NC‐1, CNF@NC‐2, CNF@NC‐3, CNF@NC‐4, CNF@NC‐6, CNF@NC‐7, respectively. Similarly, the CNF@MnO was synthesized via direct annealing of CNF@MnO_2_.

### Material Characterization

All samples were measured via powder X‐ray diffraction (XRD) using a D/Max‐2500/PC X‐ray diffractometer with Cu Ka radiation (λ = 1.5418 Å) over an angle (2θ) range from 5 to 80° at a scanning rate of 5°min^−1^. Raman spectra were recorded on a RenishawinVia laser Raman spectrometer with a laser excitation of 532 nm. The morphology and microstructure of the samples were characterized using Field‐emission Scanning Electron Microscopy (FESEM, Zeiss SUPRA 55), High‐Resolution Scanning Electron Microscope (HRSEM, SU8200), Transmission Electron Microscopy (TEM, Hitachi‐7700) and High‐Resolution Transmission Electron Microscopy (HRTEM, FEI TALOS F200) combined with High‐angle annular dark field‐scanning transmission electron microscopy (HAADF‐STEM) and energy‐dispersive X‐ray spectroscopy (EDS). N_2_ adsorption‐desorption was tested on Micromeritics ASAP 2020. The specific surface area and the pore size distribution were calculated by the Brunauer‐Emmett‐Teller (BET) and the Barrett‐Joyner‐Halenda (BJH) methods. The valence states and surface element distribution of samples were studied by X‐ray photoelectron spectroscopy (XPS) measurement on a Thermo ESCALAB 250XI. All XPS spectra were corrected using the C 1s peak (284.8 eV) as a reference. For the in situ XRD and Raman tests, the working electrodes were made by mixing the active materials (90 wt.%, CNF@NC‐5) and carboxymethyl cellulose sodium (CMC, 10 wt.%) in a mortar using deionized water as solvent. The aluminum mesh was used as the collector, performed at preselected potentials during the first discharge and charge process at a current density of 0.1 A g^−1^, and was tested on the LAND‐CT2001A.

### Electrochemical Measurements

The K‐ion storage performance of samples was evaluated by assembling the CR2032 coin cells. The working electrodes were made by mixing the active materials (70 wt.%), conductivity agent (Super‐P, 20 wt.%), and carboxymethyl cellulose sodium (CMC, 10 wt.%) in a mortar using deionized water as solvent. The well‐mixed slurry was coated onto Cu foil using a doctor blade dried in a vacuum oven at 70 °C for 3 h, and then cut into Φ12 mm disks. The mass loading density of the electrodes was controlled at 1.0 ± 0.1 mg cm^−2^. Then, the cells were assembled in an argon‐filled glove box (H_2_O, O_2_ << 1 ppm). Home‐made potassium metal foil served as both the counter and reference electrode, Whatman GF/D glass fiber was employed as the separator, and 1 M KFSI dissolved in a mixture of ethylene carbonate (EC) and diethyl carbonate (DEC) (V/V,1:1) was acted as the electrolyte. Assembled cells were aged overnight before testing. Galvanostatic charge–discharge (GCD) profiles were collected on the LAND‐CT2001A battery‐testing instrument at 25 °C between 0.001 and 3.0 V versus K^+^/K. Cyclic voltammetry (CV) measurements were conducted on an electrochemical workstation (CHI 660E, Shanghai Chenhua, China) with a scanning rate of 0.5 mV s^−1^ between 0.001 and 3.0 V versus K^+^/K. Electrochemical impedance spectroscopy (EIS, CHI 660E) tests were conducted with a frequency range from 10^5^ Hz to 0.01 Hz. In situ EIS analysis was performed at preselected potentials during the first discharge and charge process at a current density of 0.1 A g^−1^. The K‐ion diffusion coefficient was analyzed via the Galvanostatic intermittent titration technique (GITT) result, which was performed galvanostatic charging/discharging for 600 s in the potential range of 0.001–3.0 V with a relaxation time of 600 s (LAND‐CT2001A). For the K‐ion full cell, perylene tetracarboxylic anhydride (PTCDA) annealed at 450 °C for 4 h was used as the cathode (80 wt.% PTCDA:10 wt.% Super P:10 wt.% CMC), coated onto carbon coated aluminum foil, with pre‐potassium in half cell. The active materials ratio of cathode/anode in PTCDA/CNF@NC‐5 full cell was ≈2:1. The coin cell type, separator, and electrolyte used in the full cell were also the same as that in half‐cell. The anodes were activated for three cycles at 0.1 A g^−1^ before assembling full cells. The electrochemical performances of the full cell were tested on the LAND‐CT2001A with a voltage window of 0.1–2.9 V. The hybrid capacitors were constructed with a CNF@NC‐5 anode and the purchased commercial activated carbon (AC) cathode with a weight ratio of 1:1. The electrochemical performances of the hybrid capacitors were tested on the LAND‐CT2001A with a voltage window of 0.01–4.0 V. The current density was based on the total active material mass (cathode and anode).

## Conflict of Interest

The authors declare no conflict of interest.

## Supporting information

Supporting Information

## Data Availability

The data that support the findings of this study are available from the corresponding author upon reasonable request.
