# Supplementary material for: In Situ Chemical Modulation of Graphitization Degree of Carbon Fibers and Its Potassium Storage Mechanism
Source: Adv Sci (Weinh). 2024 Apr 1;11(23):2401292. doi: 10.1002/advs.202401292 (PMC11187913; doi:10.1002/advs.202401292)
Supplement: Supplementary file 1 — Supporting Information [file ADVS-11-2401292-s001.pdf]

ADVANCED SCIENCE
Open Access

Supporting Information

for *Adv. Sci.*, DOI 10.1002/advs.202401292

In Situ Chemical Modulation of Graphitization Degree of Carbon Fibers and Its Potassium Storage Mechanism

Shuangsheng Xiong, Qi Wu, Yuan Gao, Zhiping Li, Chen Wang, Shuo Wang, Zheng Li, Li Hou* and Faming Gao**

Supporting information

In Situ Chemical Modulation of Graphitization Degree of Carbon Fibers and Its Potassium Storage Mechanism

Shuangsheng Xiong[1], Qi Wu[1],Yuan Gao1, Zhiping Li[1, *], Chen Wang [1], Shuo Wang[1], Zheng Li[1] , Li Hou[1, *] and Faming Gao[1,2, *]

[1] Hebei Key Laboratory of Applied Chemistry, State Key Laboratory of Metastable Materials Science and Technology, Yanshan University, Qinhuangdao 066004, China

[2] College of Chemical Engineering and Materials science, Tianjin University of Science and Technology, Tianjin 300457, China

* Corresponding authors.

E-mail address: zpli@ysu.edu.cn; holy@ysu.edu.cn; fmgao@ysu.edu.cn

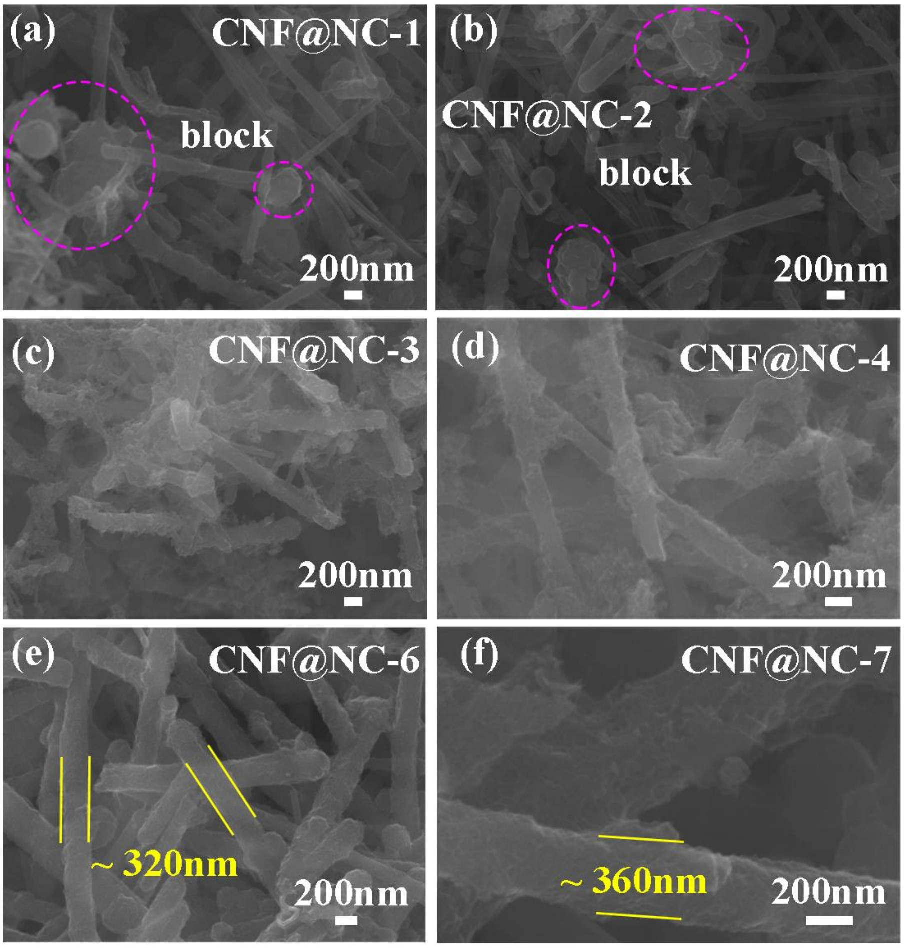

Figure S1. SEM images of samples: a) CNF@NC-1, b) CNF@NC-2, c) CNF@NC-3, d) CNF@NC-4, e) CNF@NC-7, and d) CNF@NC-7.

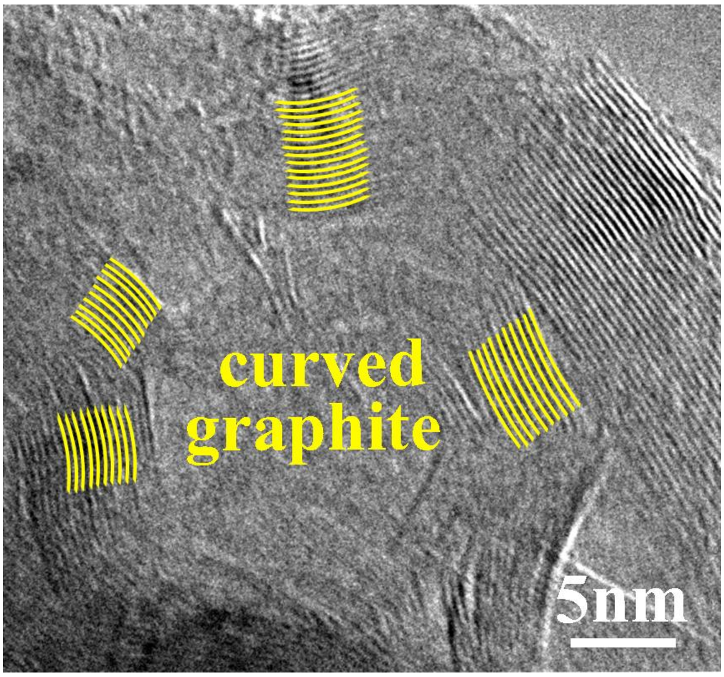

Figure S2. The HRTEM image of CNF@NC-5.

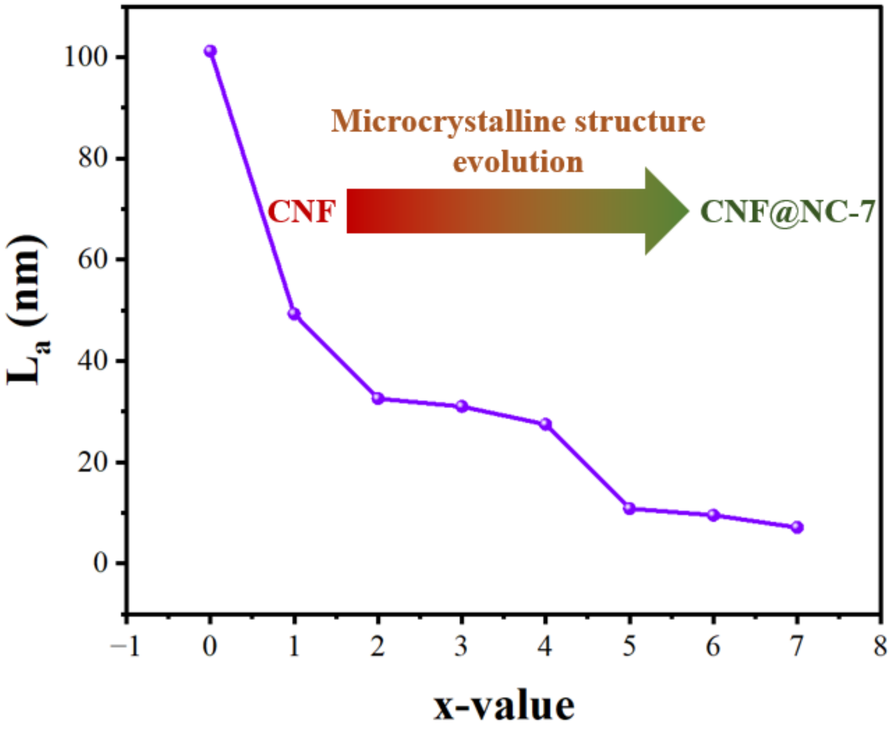

Figure S3. The change of L_a with x-value (CNF and CNF@NC-x (x = 1, 2, 3, 4, 5, 6, and 7)).

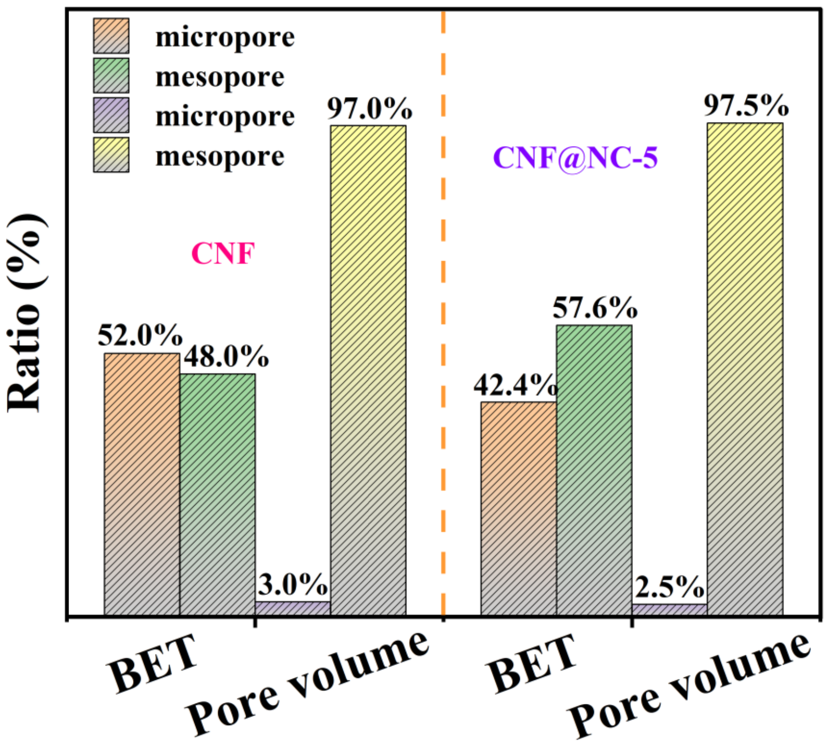

Figure S4. The ratio of mesoporous and microporous for Brunauer-Emmett-Teller and pore volume of CNF and CNF@NC-5.

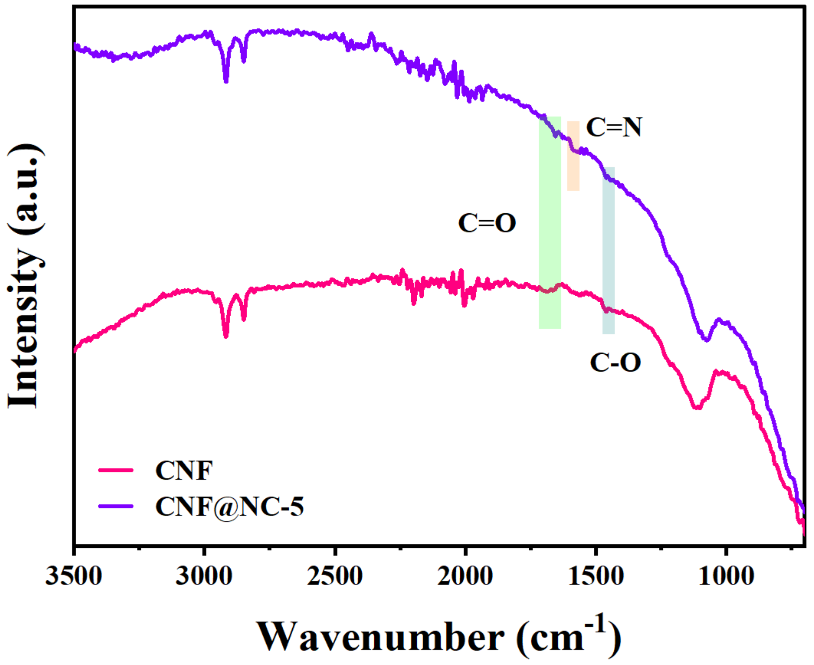

Figure S5. FTIR spectra of CNF and CNF@NC-5.

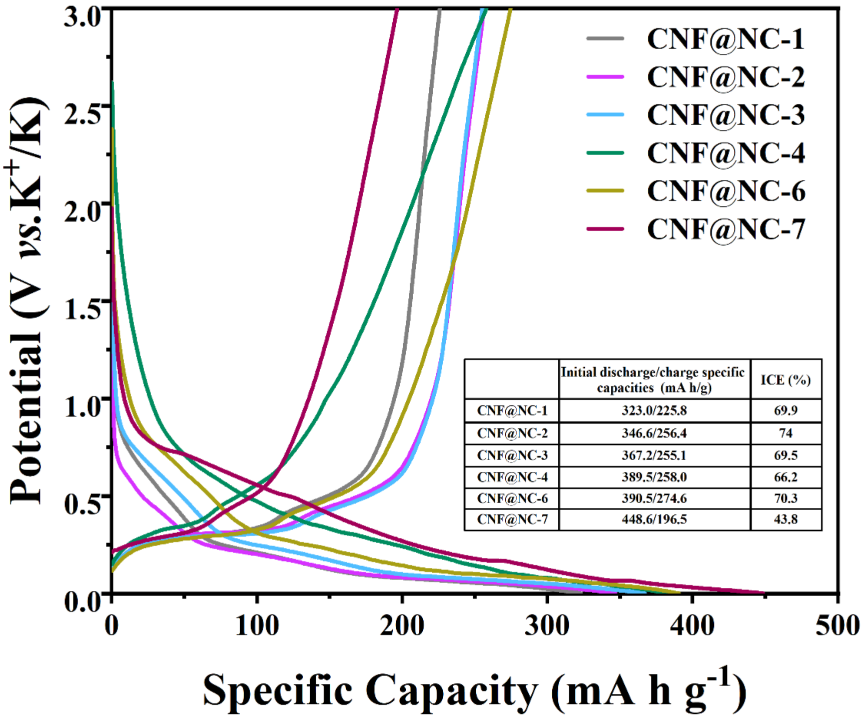

Figure S6. The first galvanostatic charge/discharge curves at 0.1 A g^{-1} of CNF@NC-x electrodes (x = 1, 2, 3, 4, 6, and 7).

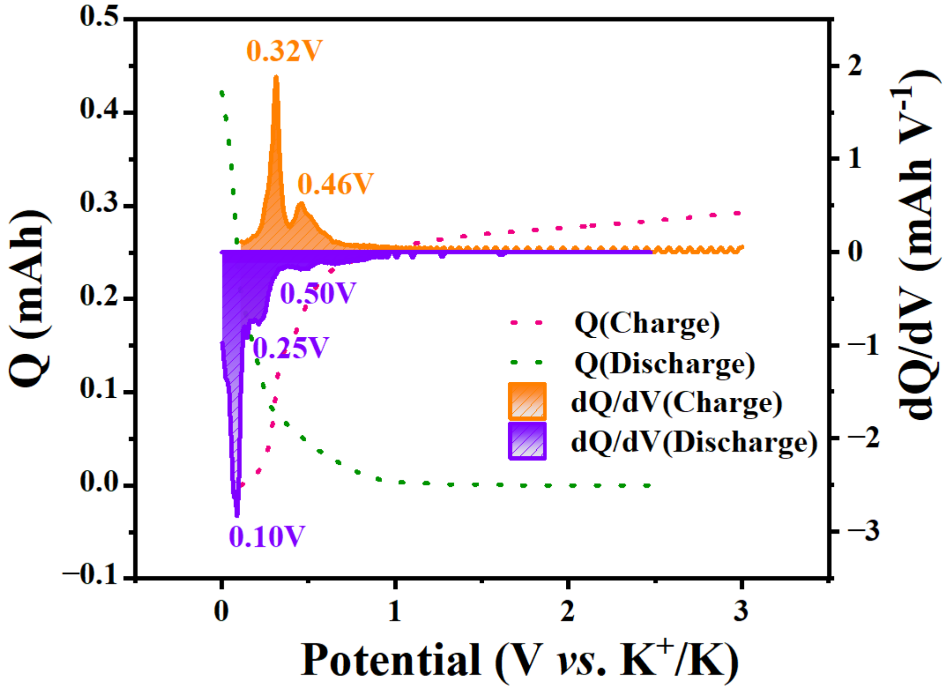

Figure S7. The dQ/dV profiles of the CNF@NC-5 electrode.

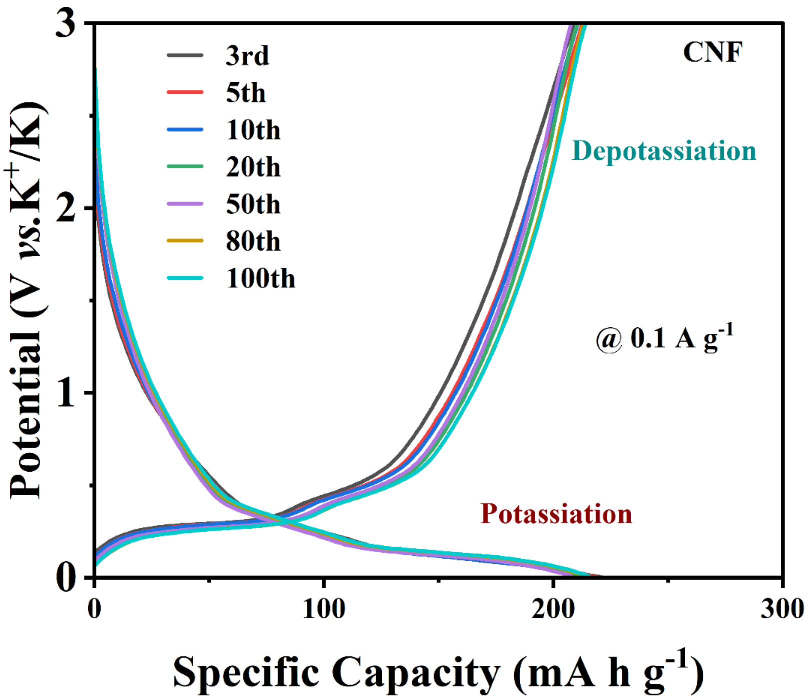

Figure S8. The charge-discharge curves for selected cycles of CNF electrode at 0.1 A g^{-1}.

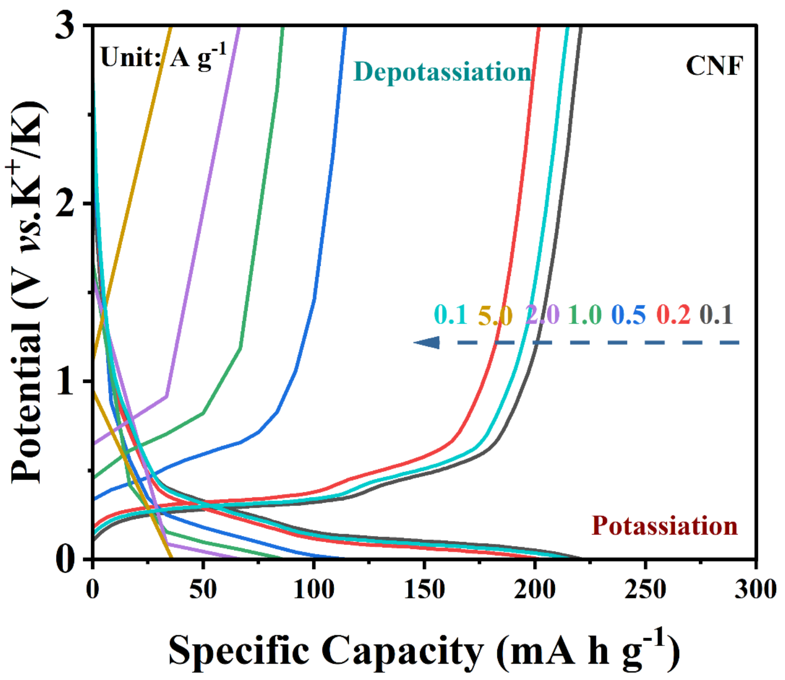

Figure S9. The charge-discharge curves at various current densities of CNF electrode (0.1, 0.2, 0.5, 1.0, 2.0, 5.0, and 0.1 A g^{-1}).

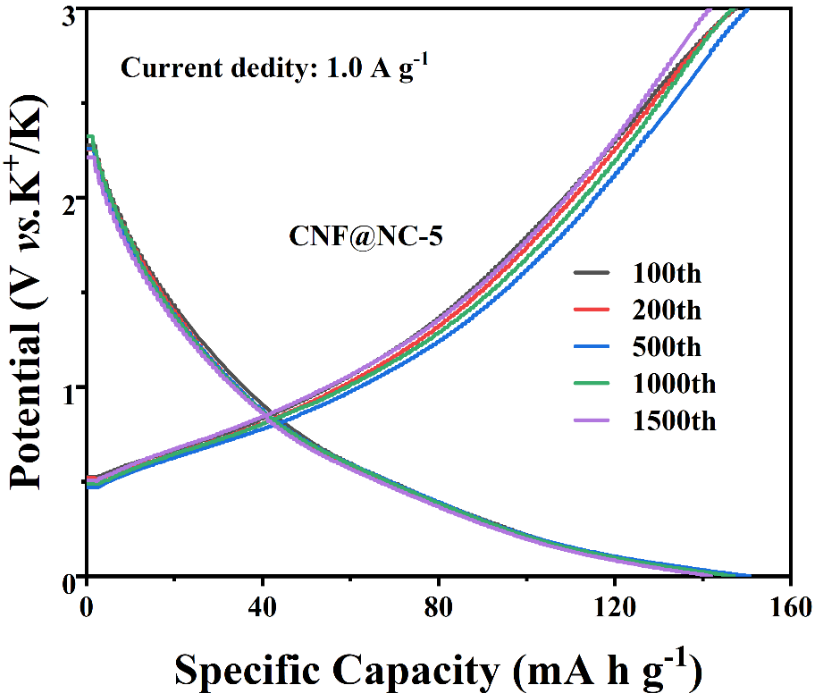

Figure S10. The charge-discharge curves for selected cycles of CNF@NC-5 electrode at 1.0 A g^{-1}.

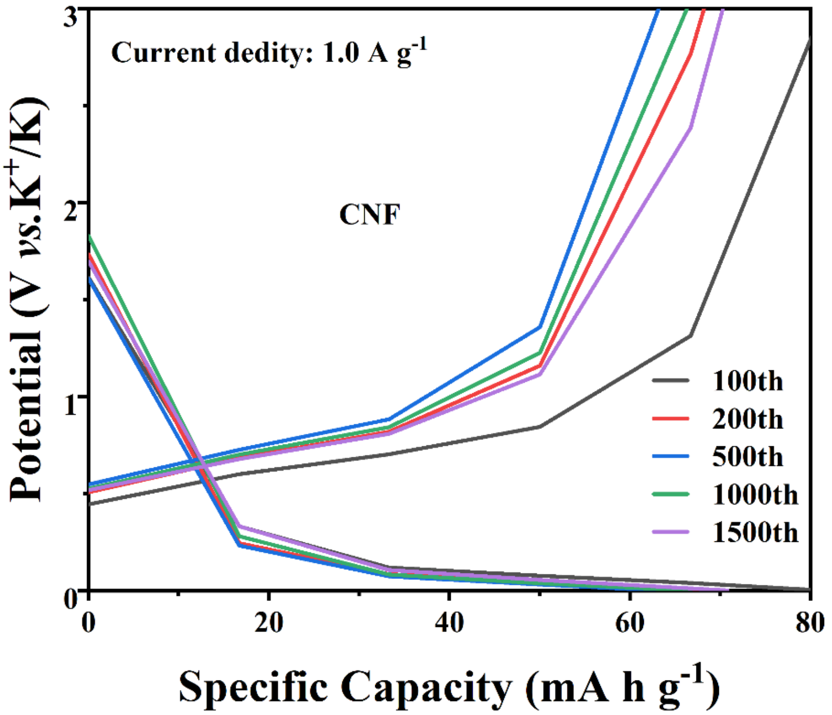

Figure S11. The charge-discharge curves for selected cycles of CNF electrode at 1.0 A g^{-1}.

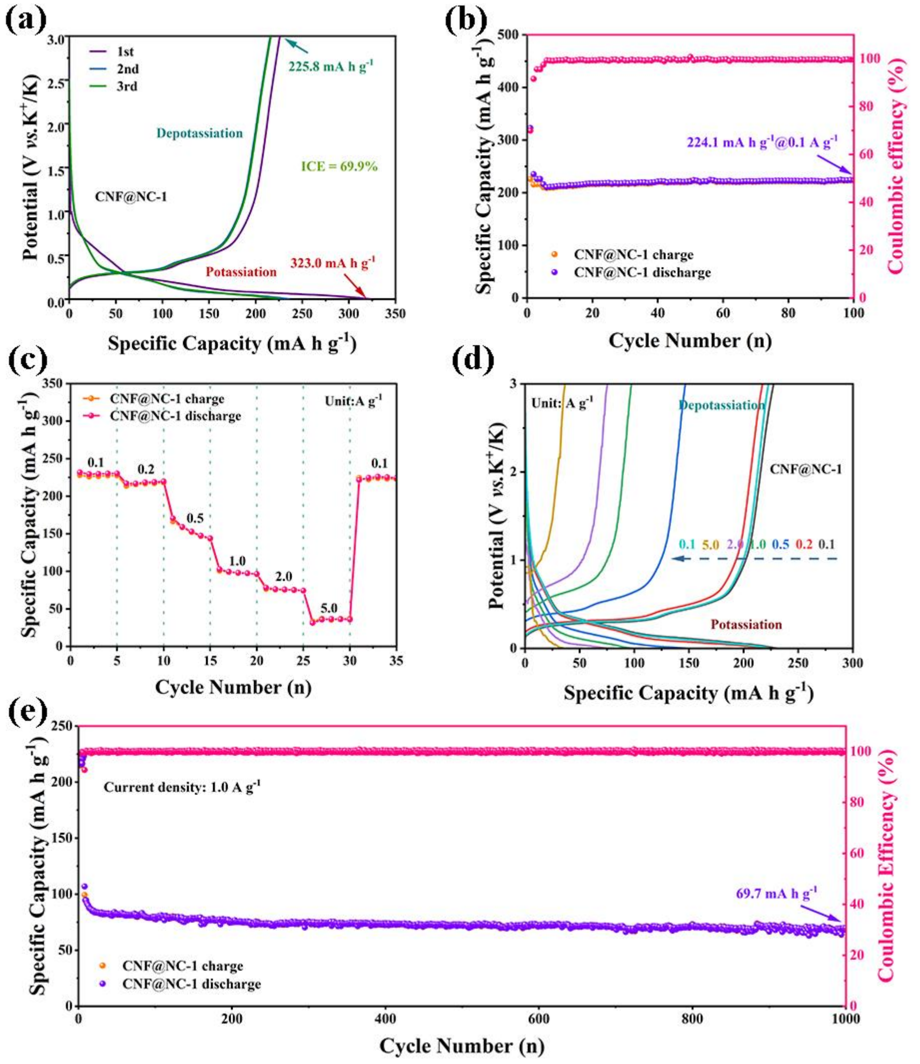

Figure S12. a) The first three galvanostatic charge/discharge curves of CNF@NC-1 electrode at 0.1 A g^{-1}. b) Potassiation and depotassiation capacity and coulombic efficiency of CNF@NC-1 electrodes at 0.1 A g^{-1}. c) The rate capability of CNF@NC-1 electrode from 0.1 to 5.0 A g^{-1}. d) The charge-discharge curves at various current densities of CNF@NC-1 electrode (0.1, 0.2, 0.5, 1.0, 2.0, 5.0, and 0.1 A g^{-1}). e) Potassiation and depotassiation capacity of CNF@NC-1 electrode at 1.0 A g^{-1}.

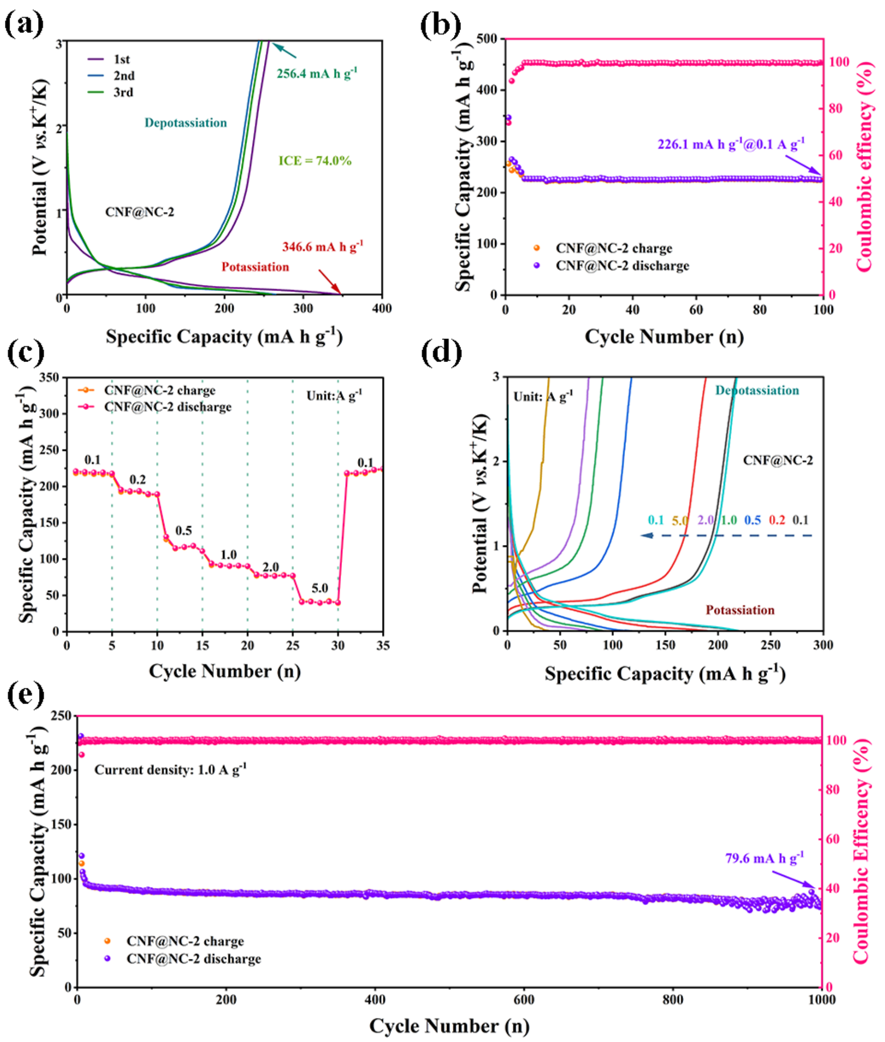

Figure S13. a) The first three galvanostatic charge/discharge curves of CNF@NC-2 electrode at 0.1 A g^{-1}. b) Potassiation and depotassiation capacity and coulombic efficiency of CNF@NC-2 electrodes at 0.1 A g^{-1}. c) The rate capability of CNF@NC-2 electrode from 0.1 to 5.0 A g^{-1}. d) The charge-discharge curves at various current densities of CNF@NC-2 electrode (0.1, 0.2, 0.5, 1.0, 2.0, 5.0, and 0.1 A g^{-1}). e) Potassiation and depotassiation capacity of CNF@NC-2 electrode at 1.0 A g^{-1}.

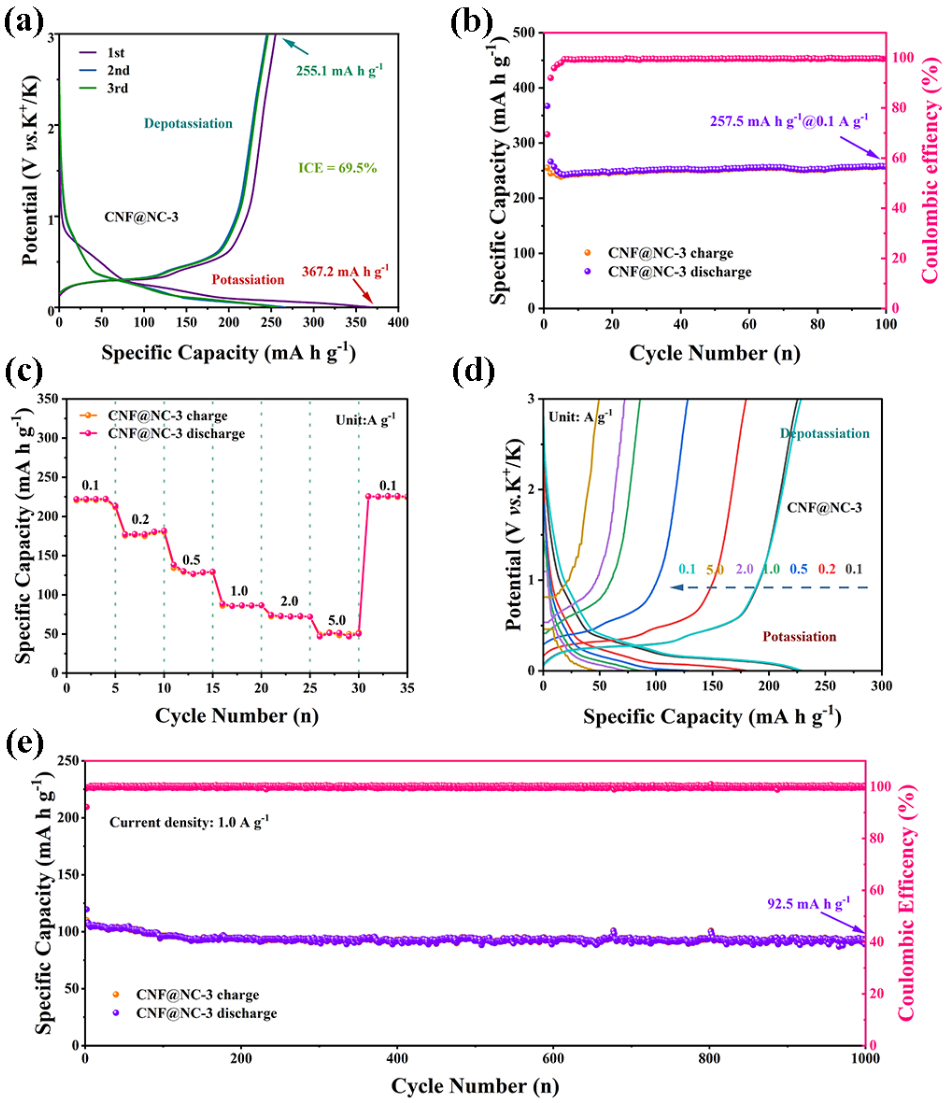

Figure S14. a) The first three galvanostatic charge/discharge curves of CNF@NC-3 electrode at 0.1 A g^{-1}. b) Potassiation and depotassiation capacity and coulombic efficiency of CNF@NC-3 electrodes at 0.1 A g^{-1}. c) The rate capability of CNF@NC-3 electrode from 0.1 to 5.0 A g^{-1}. d) The charge-discharge curves at various current densities of CNF@NC-3 electrode (0.1, 0.2, 0.5, 1.0, 2.0, 5.0, and 0.1 A g^{-1}). e) Potassiation and depotassiation capacity of CNF@NC-3 electrode at 1.0 A g^{-1}.

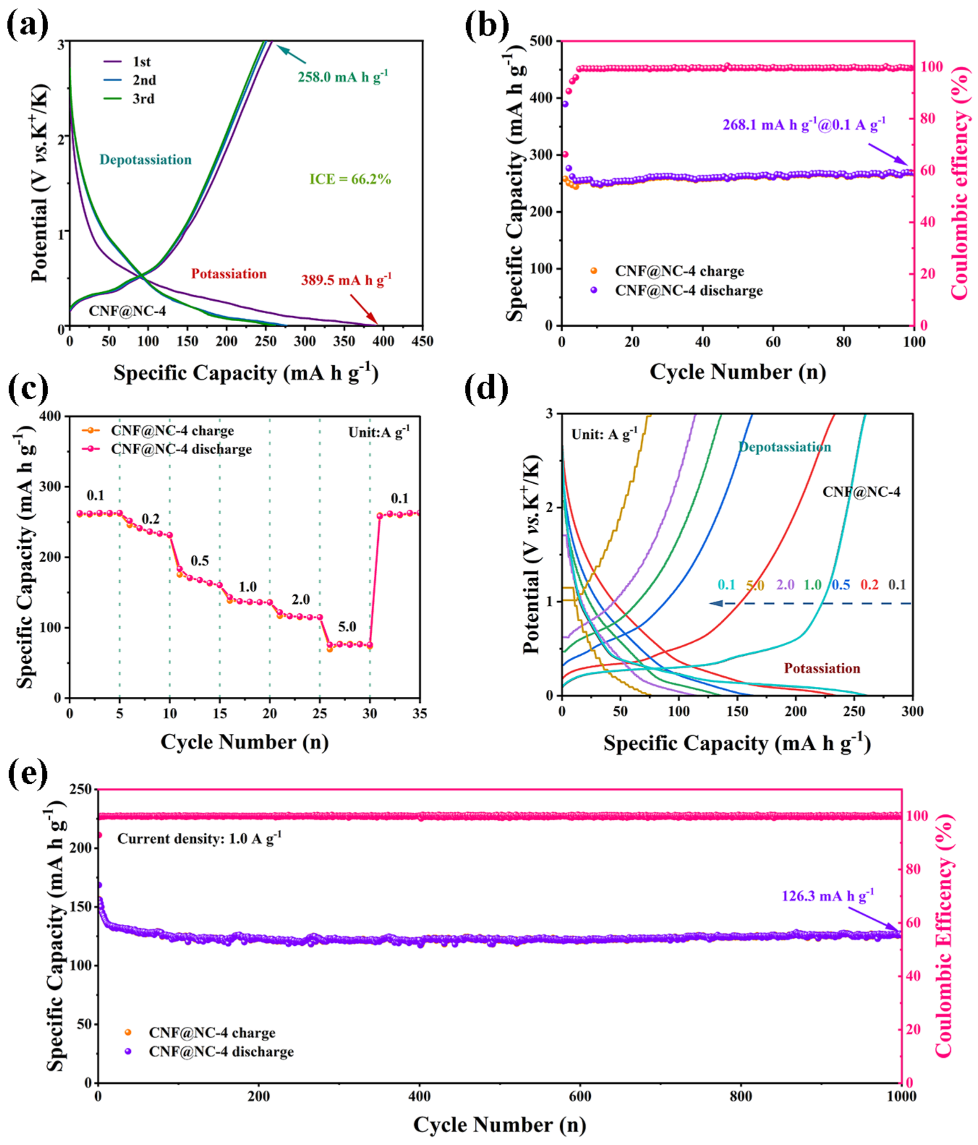

Figure S15. a) The first three galvanostatic charge/discharge curves of CNF@NC-4 electrode at 0.1 A g^{-1}. b) Potassiation and depotassiation capacity and coulombic efficiency of CNF@NC-4 electrodes at 0.1 A g^{-1}. c) The rate capability of CNF@NC-4 electrode from 0.1 to 5.0 A g^{-1}. d) The charge-discharge curves at various current densities of CNF@NC-4 electrode (0.1, 0.2, 0.5, 1.0, 2.0, 5.0, and 0.1 A g^{-1}). e) Potassiation and depotassiation capacity of CNF@NC-4 electrode at 1.0 A g^{-1}.

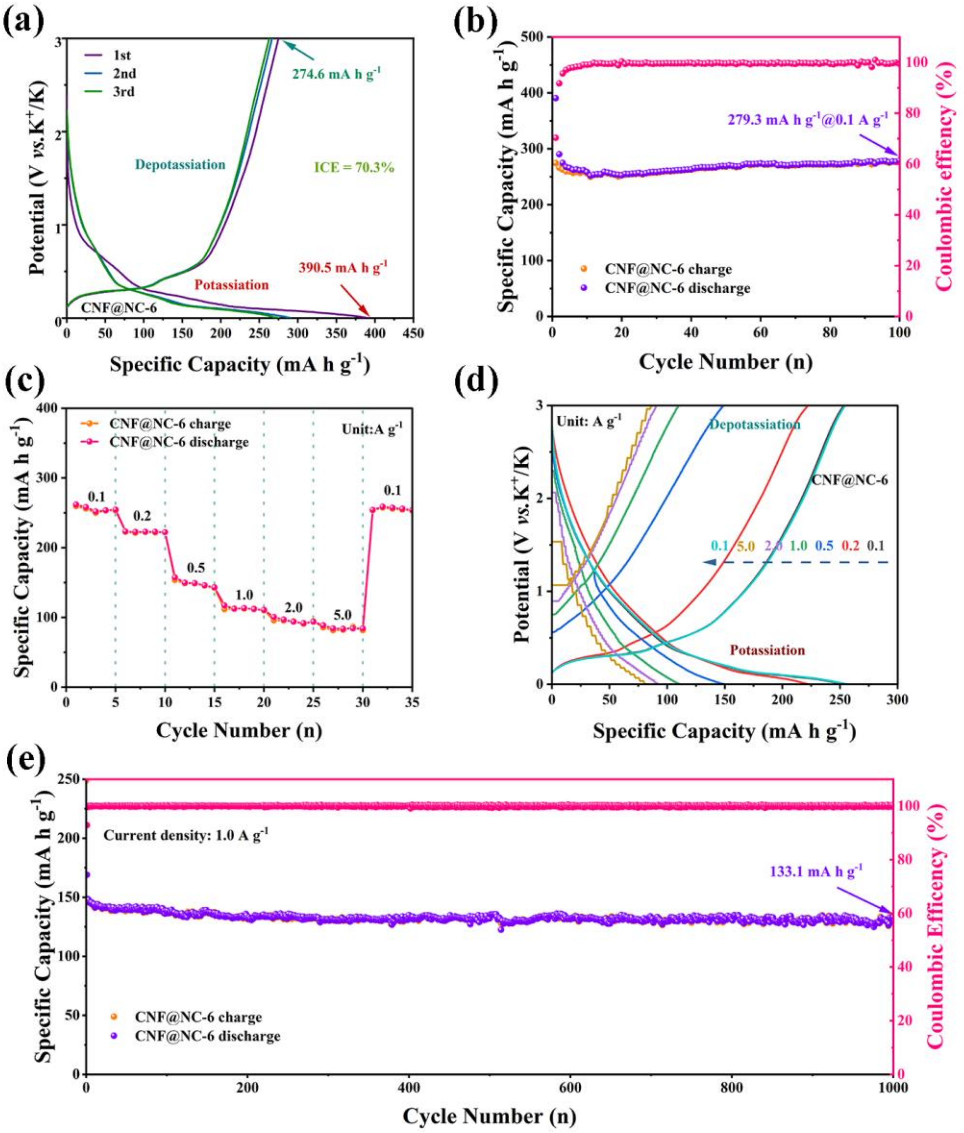

Figure S16. a) The first three galvanostatic charge/discharge curves of CNF@NC-6 electrode at 0.1 A g^{-1}. b) Potassiation and depotassiation capacity and coulombic efficiency of CNF@NC-6 electrodes at 0.1 A g^{-1}. c) The rate capability of CNF@NC-6 electrode from 0.1 to 5.0 A g^{-1}. d) The charge-discharge curves at various current densities of CNF@NC-6 electrode (0.1, 0.2, 0.5, 1.0, 2.0, 5.0, and 0.1 A g^{-1}). e) Potassiation and depotassiation capacity of CNF@NC-6 electrode at 1.0 A g^{-1}.

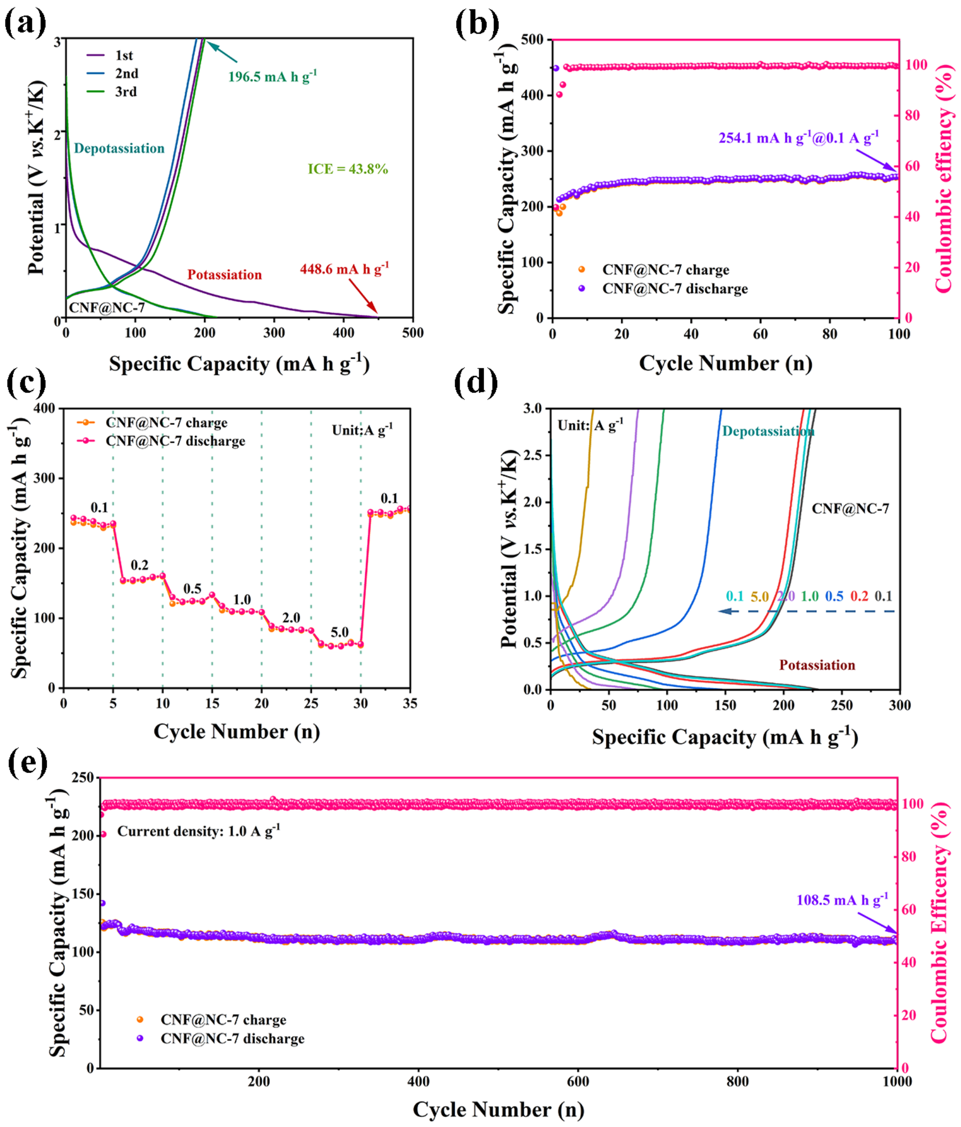

Figure S17. a) The first three galvanostatic charge/discharge curves of CNF@NC-7 electrode at 0.1 A g^{-1}. b) Potassiation and depotassiation capacity and coulombic efficiency of CNF@NC-7 electrodes at 0.1 A g^{-1}. c) The rate capability of CNF@NC-7 electrode from 0.1 to 5.0 A g^{-1}. d) The charge-discharge curves at various current densities of CNF@NC-7 electrode (0.1, 0.2, 0.5, 1.0, 2.0, 5.0, and 0.1 A g^{-1}). e) Potassiation and depotassiation capacity of CNF@NC-7 electrode at 1.0 A g^{-1}.

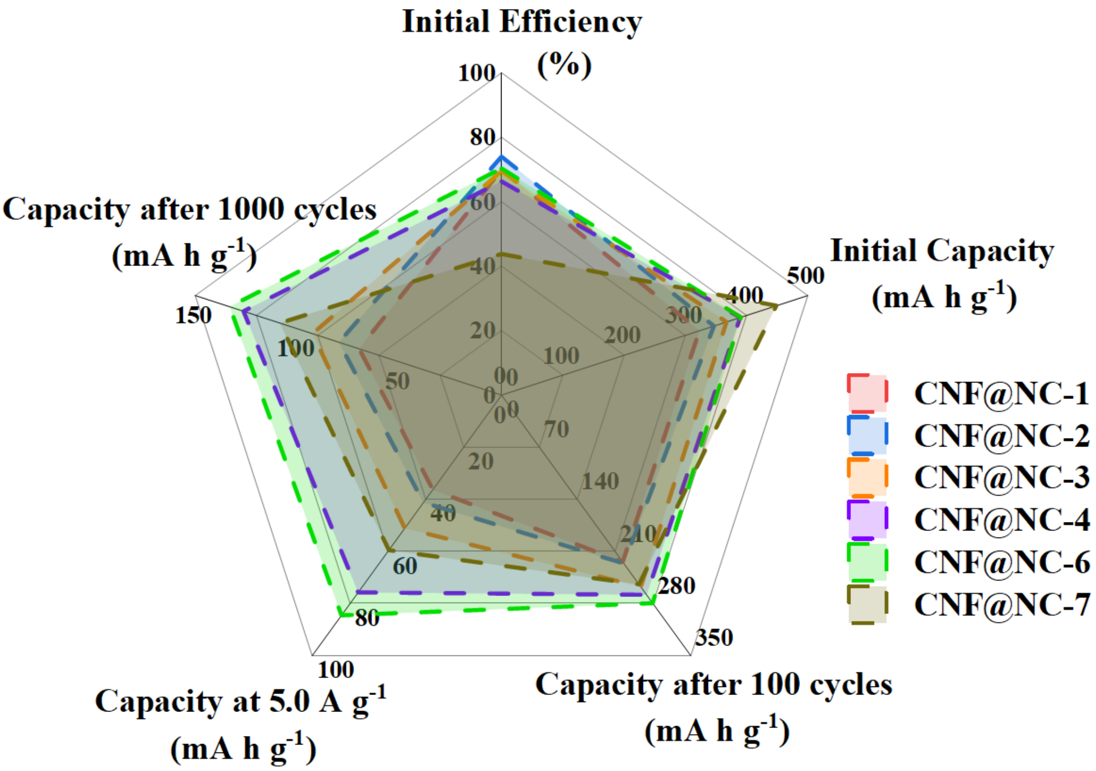

Figure S18. Comparison of all aspects of CNF@NC-x (x = 1, 2, 3, 4, 6, and 7).

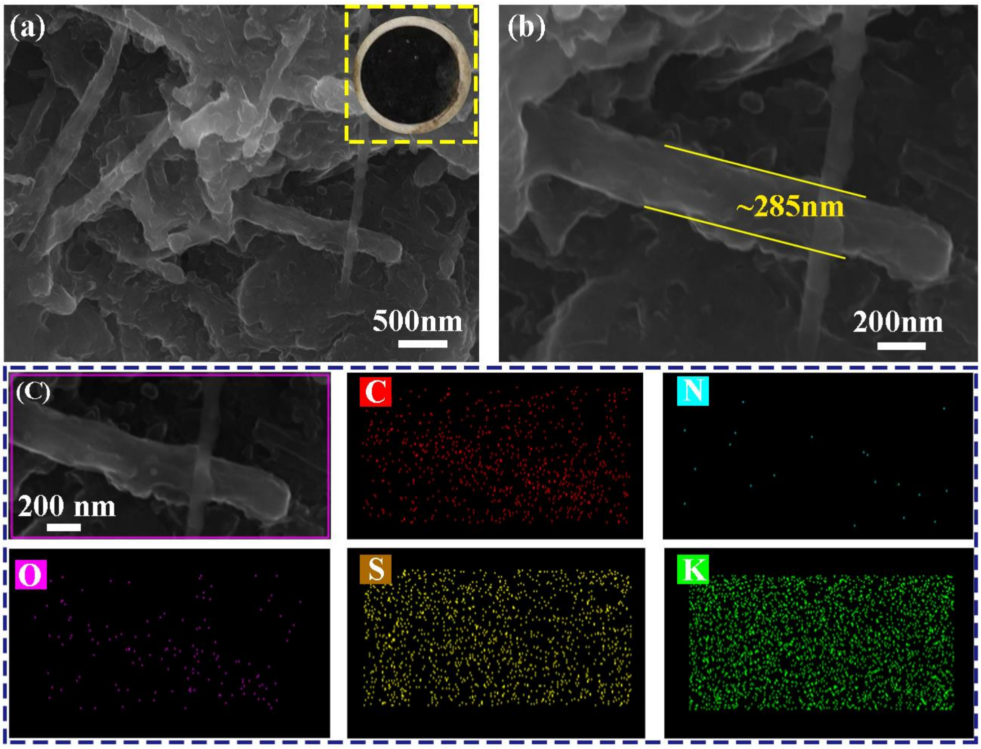

Figure S19. The CNF@NC-5 electrode after 2000 cycles: a) low-magnification and b) high-magnification SEM images; c) the corresponding EDS mapping images.

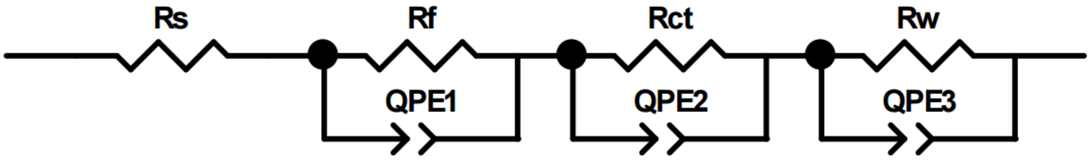

Figure S20. An equivalent circuit for EIS analysis.

The R_s, R_f, R_{ct}, and R_W signified the internal resistance, the electrolyte/SEI interface resistance, the charge-transfer resistance, and the Warburg resistance, respectively. [1] The diffusion coefficient of K^+ could be calculated by the following formula:

$$D_K = \frac{1}{2}\left(\frac{RT}{An^2F^2C\sigma'}\right)^2$$

In this formula, D_K was the diffusion coefficient of K^+, R was the gas constant (8.314 J mol^{-1} K^{-1}), T was the test temperature of battery (298 K), F was the Faraday constant (96500 C mol^{-1}), A was the surface area of electrode (1.13 cm^{-2}), n was the number of electrons involved in the insertion reaction (n = 1), C was the K^+ concentration in electrode (C = 1.0×10^{-3} mol cm^{-1}) and σ' was the Warburg coefficient equaled to the slopes of the Z'(Ω) and $\omega^{-1/2}$ (ω = 2πf) lines in the low-frequency region.[2]

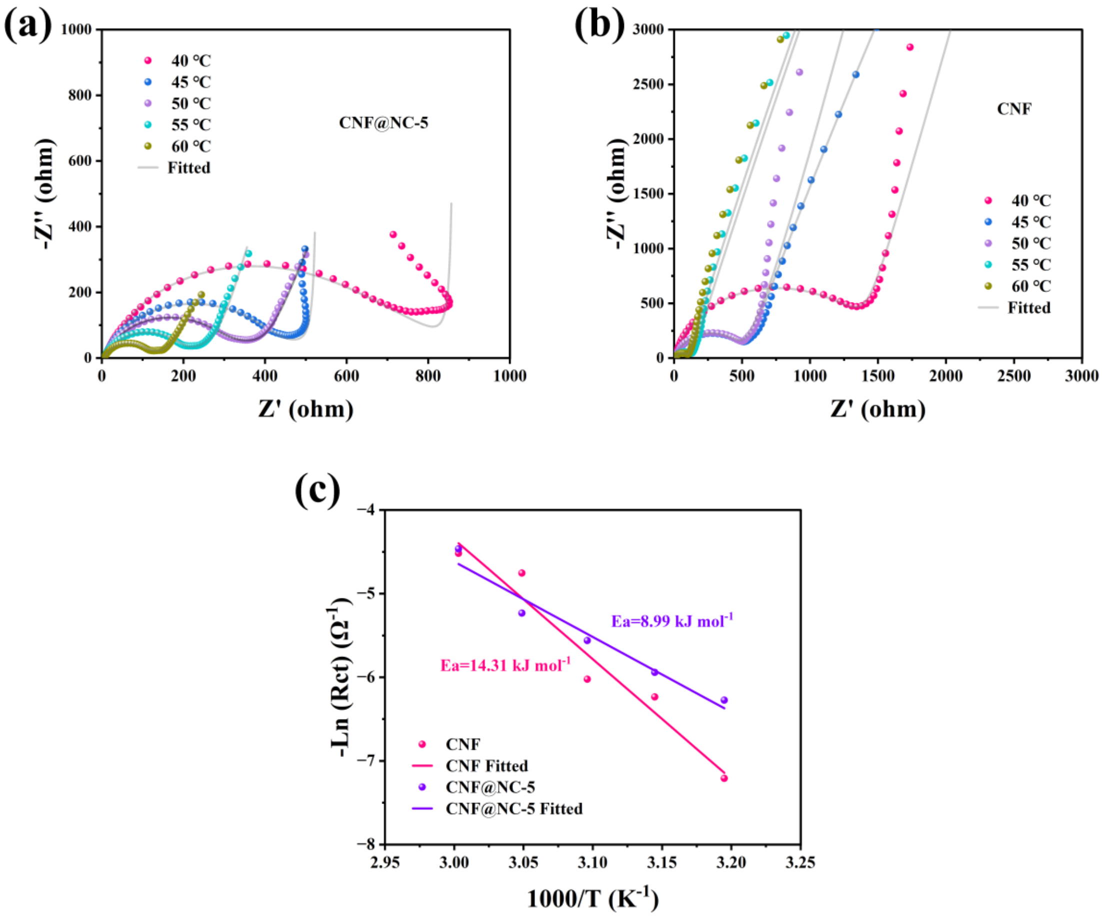

Figure S21. EIS measurement at various temperatures of a) CNF@NC-5 electrode, and b) CNF electrode. c) The calculation of activation energy of CNF and CNF@NC-5 electrodes.

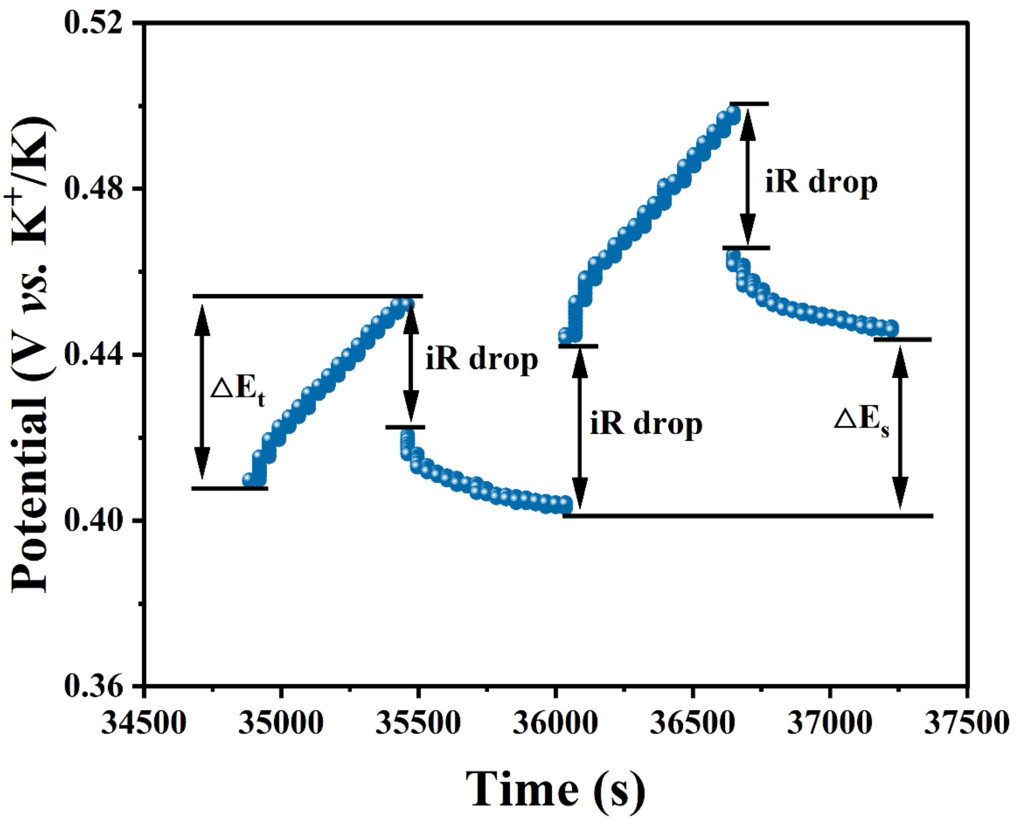

Figure S22. Determination of ΔE_t and ΔE_s from the measured GITT profiles.

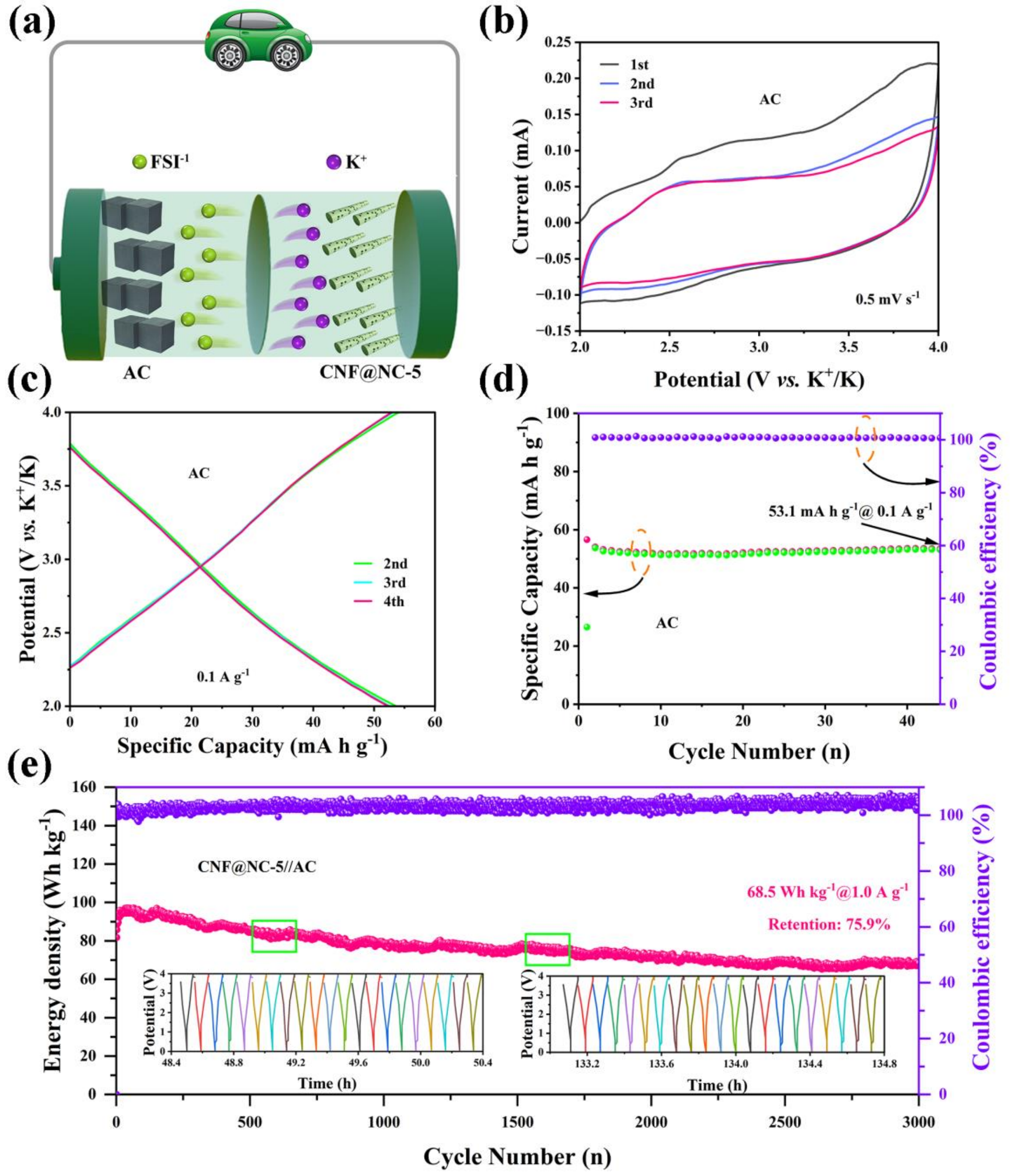

Figure S23. CNF@NC-5//AC PIHCs. a) Diagram of PIHCs. b) CV profiles of AC in half cells between 2.0 and 4.0 V vs K^+/K at 0.5 mV s^{-1}. c) The 2nd, 3rd, and 4th charge-discharge curves at 0.1 A g^{-1} of AC in half cells. d) Potassiation and depotassiation capacity and coulombic efficiency at 0.1 A g^{-1} of AC in half cells. e) Long-term cycle performance of CNF@NC-5//AC PIHCs at 1.0 A g^{-1} over 3000 cycles.

Table S1. The volumetric specific capacity of reported carbon materials in KIBs.

Sample	Current density: A g^{-1}	Capacity: mA h cm^{-3}	Cycle number	Reference
**CNF@NC-5**	**0.1**	**510.2**	**100**	**This work**
**CNF**	**0.1**	**390.1**	**100**	
**GNFM-1.5**	0.05	316.3	100	[3]
**GNFM-3**		298.0		
**GNFM-5**		287.0		
**GNFM-10**		285.7		
**GNF-PVDF-1.5**		255.8		
**GNF-PVDF-3**		209.6		
**GNF-PVDF-5**		165.4		
**BCNBs**	0.05	426.9	-	[1]
**EG**	0.01	473.4	-	[4]

Table S2. Electrochemical performance of reported carbon materials in KIBs.

Sample	ICE at A g^{-1}	Current density: A g^{-1}	Capacity: mA h g^{-1}	Cycle number	Reference
**CNF@NC-5**	**69.3% at 0.1 A g^{-1}**	**0.1**	**294.9**	**100**	**This work**
		**1.0**	**141.9**	**1500**	
		**2.0**	**98.3**	**2000**	
graphite	62.9% at 0.5 A g^{-1}	2.0	~ 90	100	[5]
S/N@C	~ 24% at 0.05 A g^{-1}	0.1	200	400	[6]
		1.0	103	300	
		2.0	65	900	
CDs@rGO	44% at 0.1 A g^{-1}	0.1	311	280	[7]
		0.2	244	840	
NOHPHC	23.9% at 0.05 A g^{-1}	0.05	230.6	100	[8]
		1.05	124.8	1100	
NGHCs-750	34.84% at 0.05 A g^{-1}	0.05	298.8	100	[9]
		0.5	137.6	1000	
C_{30}	30.1% at 0.2 A g^{-1}	0.2	221.2	100	[10]
		4.0	104.9	1350	
CNC	40% at 0.0558 A g^{-1}	0.0558	195	100	[11]

OGCS	61.2% at 0.05 A g^{-1}	0.05	361.7	100	[12]
		0.5	270.8	500	
		1.0	223.6	2000	
		2.0	170	10000	
C-2100	51.8% at 0.03 A g^{-1}	0.03	292.0	100	[13]
HGC	26% at 0.05 A g^{-1}	0.05	269	200	[14]
PCMs	61.7% at 0.05 A g^{-1}	0.05	226.6	100	[15]
		0.2	201	200	
		0.5	173	200	
		1.0	108.4	2000	
CNFM-1.5	63.1% at 0.05 A g^{-1}	0.05	253.8	100	[3]
3DNFC	24.3% at 0.1 A g^{-1}	2.0	137	1000	[16]

Reference

[1] H. Liang, Z. Sun, M. Zhang, W. Hu, J. Shi, J. Chen, W. Tian, M. Huang, J. Wu, H. Wang, *Energy Environ. Mater.* **2023**, *6*, e12559, https://doi.org/10.1002/eem2.12559.

[2] S. Xiong, Y. Jiang, W. Liang, S. Deng, Y. Wang, S. Luan, R. Chen, L. Hou, Z. Zhang, F. Gao, *ChemElectroChem* **2021**, *8*, 3767, https://doi.org/10.1002/celc.202100664.

[3] B. Cao, H. Liu, P. Zhang, N. Sun, B. Zheng, Y. Li, H. Du, B. Xu, *Adv. Funct. Mater.* **2021**, *31*, 2102126, https://doi.org/10.1002/adfm.202102126.

[4] Y. An, H. Fei, G. Zeng, L. Ci, B. Xi, S. Xiong, J. Feng, *J. Power Sources* **2018**, *378*, 66, https://doi.org/10.1016/j.jpowsour.2017.12.033.

[5] L. Li, L. Liu, Z. Hu, Y. Lu, Q. Liu, S. Jin, Q. Zhang, S. Zhao, S. L. Chou, *Angew. Chem., Int. Ed.* **2020**, *59*, 12917, https://doi.org/10.1002/anie.202001966.

[6] A. Mahmood, S. Li, Z. Ali, H. Tabassum, B. Zhu, Z. Liang, W. Meng, W. Aftab, W. Guo, H. Zhang, M. Yousaf, S. Gao, R. Zou, Y. Zhao, *Adv. Mater.* **2018**, *31*, 1805430, https://doi.org/10.1002/adma.201805430.

[7] E. Zhang, X. Jia, B. Wang, J. Wang, X. Yu, B. Lu, *Adv. Sci.* **2020**, *7*, 2000470, https://doi.org/10.1002/advs.202000470.

[8] J. Yang, Z. Ju, Y. Jiang, Z. Xing, B. Xi, J. Feng, S. Xiong, *Adv. Mater.* **2017**, *30*, 1700104, https://doi.org/10.1002/adma.201700104.

[9] J. Hu, Y. Xie, M. Yin, Z. Zhang, *J. Energy Chem.* **2020**, *49*, 327, https://doi.org/10.1016/j.jechem.2020.03.005.

[10] C. Yuxiang, X. Shi, B. Lu, J. Zhou, *Adv. Energy Mater.* **2022**, *12*, 2202851, https://doi.org/10.1002/aenm.202202851.

[11] B. Cao, Q. Zhang, H. Liu, B. Xu, S. Zhang, T. Zhou, J. Mao, W. K. Pang, Z. Guo, A. Li, J. Zhou, X. Chen, H. Song, *Adv. Energy Mater.* **2018**, *8*, 1801149, https://doi.org/10.1002/aenm.201801149.

[12] Y. Qian, S. Jiang, Y. Li, Z. Yi, J. Zhou, J. Tian, N. Lin, Y. Qian, *Angew. Chem., Int. Ed.* **2019**, *58*, 18108, https://doi.org/10.1002/anie.201912287.

[13] S. H. Choi, J. Baucom, X. Li, L. Shen, Y.-H. Seong, I. S. Han, Y. J. Choi, Y. N. Ko, H. J. Kim, Y. Lu, *J. Colloid Interface Sci.* **2020**, *577*, 48, https://doi.org/10.1016/j.jcis.2020.05.051.

[14] Y. Feng, S. Chen, D. Shen, J. Zhou, B. Lu, *Energy Environ. Mater.* **2020**, *4*, 451, https://doi.org/10.1002/eem2.12126.

[15] M. Chen, W. Wang, X. Liang, S. Gong, J. Liu, Q. Wang, S. Guo, H. Yang, *Adv. Energy Mater.* **2018**, *8*, 1800171, https://doi.org/10.1002/aenm.201800171.

[16] B. Yang, J. Chen, L. Liu, P. Ma, B. Liu, J. Lang, Y. Tang, X. Yan, *Energy Storage Mater.* **2019**, *23*, 522, https://doi.org/10.1016/j.ensm.2019.04.008.
